# Can Observation Skills of Citizen Scientists Be Estimated Using Species Accumulation Curves?

**DOI:** 10.1371/journal.pone.0139600

**Published:** 2015-10-09

**Authors:** Steve Kelling, Alison Johnston, Wesley M. Hochachka, Marshall Iliff, Daniel Fink, Jeff Gerbracht, Carl Lagoze, Frank A. La Sorte, Travis Moore, Andrea Wiggins, Weng-Keen Wong, Chris Wood, Jun Yu

**Affiliations:** 1 Cornell Lab of Ornithology, Cornell University, Ithaca, New York, United States of America; 2 British Trust for Ornithology, Thetford, Norfolk, England, United Kingdom; 3 School of Information, University of Michigan, Ann Arbor, Michigan, United States of America; 4 School of Electrical Engineering and Computer Science, Oregon State University, Corvallis, Oregon, United States of America; 5 College of Information Studies, University of Maryland, College Park, Maryland, United States of America; University of Bologna, ITALY

## Abstract

Volunteers are increasingly being recruited into citizen science projects to collect observations for scientific studies. An additional goal of these projects is to engage and educate these volunteers. Thus, there are few barriers to participation resulting in volunteer observers with varying ability to complete the project’s tasks. To improve the quality of a citizen science project’s outcomes it would be useful to account for inter-observer variation, and to assess the rarely tested presumption that participating in a citizen science projects results in volunteers becoming better observers. Here we present a method for indexing observer variability based on the data routinely submitted by observers participating in the citizen science project eBird, a broad-scale monitoring project in which observers collect and submit lists of the bird species observed while birding. Our method for indexing observer variability uses species accumulation curves, lines that describe how the total number of species reported increase with increasing time spent in collecting observations. We find that differences in species accumulation curves among observers equates to higher rates of species accumulation, particularly for harder-to-identify species, and reveals increased species accumulation rates with continued participation. We suggest that these properties of our analysis provide a measure of observer skill, and that the potential to derive post-hoc data-derived measurements of participant ability should be more widely explored by analysts of data from citizen science projects. We see the potential for inferential results from analyses of citizen science data to be improved by accounting for observer skill.

## Introduction

Increasingly volunteers are recruited to participate in projects that collect observations for scientific research. The success of these “citizen science” efforts requires a balance between public recruitment [1,2] and gathering data that are able to meet scientific research objectives [3]. For example, to ensure broad participation in citizen science projects that gather observations of organisms data collection protocols often have few restrictions, which limits opportunities to control for known sources of bias (e.g., when, where and how individuals make observations) during data collection. The resulting tension between recruitment and research objectives leads to tradeoffs between data quantity and data quality [4], which has generated questions on the scientific merit of monitoring data gathered in citizen science projects [5,6]. Since data collection is often open-ended it is imperative to find ways of identifying and controlling for sources of variation or bias in the analysis [7].

A source of bias in citizen science is the variation in observer ability to detect and classify organisms to species is a potential factor in any data collected by multiple observers [8], and may be a critical issue in citizen science datasets that encompass a wide range of participants [9]. This is because every participant in a citizen science project brings their own unique levels of expertise, and the range can extend from individuals who can identify many species correctly to those who can only identify the most common and conspicuous species. Not accounting for observer variability results in systematic biases that impact analyses and interpretations. For example, observer differences can affect the detection of species that occur at low-density levels [10], and can lead to overly optimistic estimates of population trends [11]. Thus, any demonstration of the utility of citizen science data requires quantifying and accounting for observer variability in detecting and identifying species.

The duration of time spent observing organisms is expected to be related to the number of species detected—observers who make observations for 1 hour would be expected to record more species than those who make observations for 15 minutes. However, the number of species detected and identified is expected to approach an asymptote over time [12], because any given area contains a finite number of species. Ecologists have long used this process to create species accumulation curves (SACs), which describe the increase in the number of species observed with increasing time spent searching and methods have been developed to extrapolate total species richness from these SACs [13,14]. In this paper we use SACs to provide information about variation in observer’s ability in detecting and identifying organisms when the given pool of species is relatively fixed. We predict that better observers accumulate new species at a faster rate, and the rate of accumulation of new species for each observer can be used as a surrogate for observer skill. Further, while it is often presumed that participation in a citizen science project leads to increased knowledge [15], this presumption has rarely been tested. A novel test for the presence of learning would be provided if individual observers increase their rates of accumulation of species with increased participation in a citizen science project. Finally, we test whether the ability to account for individual differences in SACs improve the overall quality of citizen science data.

We explore the use of SACs to quantify observer variability using data from the citizen science project eBird [16]. We model the expected number of bird species on eBird checklists as a function of search effort to describe the accumulation of species across all observers’ data. In each of six study regions that represent different pools of potentially observable species, we statistically control for several factors we expect to have additional effects on the available pool of species (e.g., season and habitat). Based on this model, we create a single numeric index the SAC index that describes the expected number of species reported by each observer, had all observers conducted the same standardized search. We use this index to: 1) explore inter-observer variation in data collection, and 2) explore whether increased ability is a simple increase in rates of detection or whether there is evidence of systematic changes in observer skill (i.e., learning) with increasing experience.

## Materials and Methods

### eBird

eBird is comprised of a network of more than 200,000 citizen science participants who have submitted more than 250 million observations from most countries of the world, and have recorded 97% of all recognized bird species. Observations are submitted to eBird in checklist format, which are lists of bird species observed at one survey location. Additionally, each checklist contains information on the location, date, start time and effort the observer expended collecting the observations on the checklist. Effort information includes both the duration and distance travelled while birding, the number of people who were in the birding party, and whether the checklist includes all of the birds that were identified (we use this last piece of information to infer absence of species) [17]. Participants submit their checklists either online or through apps on handheld devises to a centralized database.

All eBird records are reviewed for accuracy through a combined process of automated filters that identify unusual records and a network of more than 900 expert reviewers who assess the accuracy of those records. The eBird data management strategy [18] links every checklist to an observer and to the location where the observations were made allowing us to analyze eBird submissions at the level of an individual contributor. For this study we used complete eBird checklists from the United States that were submitted between 2004–2012, inclusive.

### Calibrating eBird Observers

This study does not directly measure individual eBird observer’s skill levels. Instead we take a data-driven approach by using eBird participant’s data submissions to allow us to infer their skill level. There are two principal facets to adding a bird onto an eBird checklist: detection and identification. Detection begins with visual or auditory cues that allow an observer to find a bird. These same auditory and visual cues are combined with knowledge of species habitat preferences and typical behaviors in a highly cognitive process to allow the observer to identify the species. With experience, both of these processes—detection and identification—become much faster, allowing experienced birders to quickly find and identify birds. Accumulating the knowledge to find and identify species quickly requires hundreds of hours of effort learning about bird identification. The result is large differences between observers in their skill level in detecting and identifying species, which is reflected in the number of species that they report on an eBird checklist. For example, some species have life history traits (i.e., come to feeders) that make them much easier to find; we expect that these species be found at more similar rates among all observers. Other species that are more cryptic (i.e., quiet or difficult to observe) are likely to be identified more frequently by more experienced birders than those with less experience.

We consider more experienced observers to be those who detect more of the species that are present (i.e., have fewer false negatives) and identify the majority of species correctly (i.e., fewer false positives). Less experienced observers can be expected to make both types of errors, in that they will miss many species that are available for detection and may also misidentify some species, leading to false positives. The existing eBird review process addresses many of the latter issues [17,19,20], while our approach described in this paper is an attempt to address the former.

**Fig 1:**
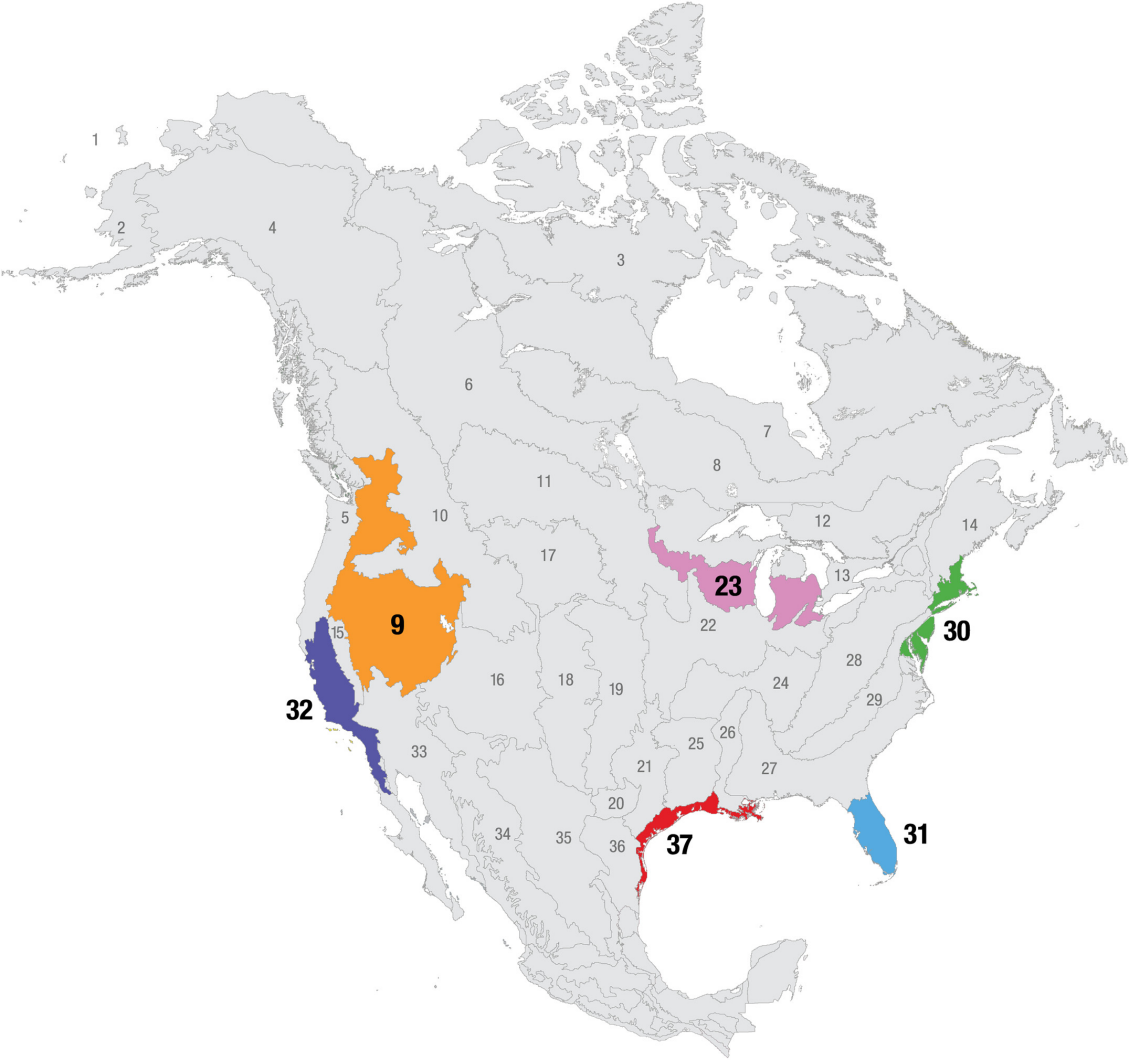
The Bird Conservation Regions (BCRs) of North America. BCRs are ecologically distinct regions with similar bird communities. BCRs were used to cluster checklists into groups with similar likelihoods of species encounter. Data from six BCRs were used in this study: BCR 9- Great Basin, BCR 23- Prairie Hardwood Transition, BCR 30- New England/Mid-Atlantic Coast, BCR 31- Peninsular Florida, BCR 32- Coastal California, and BCR 37- Gulf Coastal Prairie. These BCRs were selected to include a range of eBird participation (Table 1) and varying degrees of diversity and patterns of bird occurrence. Map provided by the North American Bird Conservation Initiative (www.nabci-us.org).

We constructed the SAC index by modeling the number of species observed on a checklist as a function of the time spent observing. We expect longer durations birding to record a greater number of species and for this rate of increase to slow with increasingly longer checklists. We expect observers with higher ability to record more species in a given period of time. However, there are numerous factors that affect which species are available for detection at a given location and time, and differences in the number of species recorded may be due to these factors, rather than observer differences. To produce the most accurate estimate of observer differences in species accumulation rates, we had to account for these other factors affecting number of species. At a geographic scale, we analyzed data separately for each of six Bird Conservation Region (BCR)—ecoregions that reflect similar bird communities, habitats and resource management issues and have been widely used for bird research and conservation [21] (Fig 1).

**Table 1:** The number of observers and number of checklists submitted for each Bird Conservation Region.

BCR	Total Number of Observers	Total Number of Checklists
**9**	1,153	76,501
**23**	2,116	131,719
**30**	3,660	312,987
**31**	1,564	72,572
**32**	2,517	196,171
**37**	945	35,755

Three categories of covariates were used to describe other factors that may affect the number of species on a checklist: 1) the number of species present within the BCR where the checklists were collected, 2) factors that affect the probability an observer would detect a species in a BCR and, 3) the observer’s ability to detect and identify species. We selected appropriate covariates to model sources of variation in categories 1 and 2 in order to produce the most accurate observer description of inter-observer variation in the rate of accumulation of new species with increased effort (category 3). This was done by fitting a generalized additive mixed model (GAMM) with the number of species observed, (i.e., the estimate of species richness), on each checklist as the response variable. We modeled the error distribution in the GAMM as Poisson using a log link function. Observer was included as a random effect. The covariates included in the model as fixed effects are described in detail below.

### Covariates Describing the Number of Species Present

The first group of covariates, describing the total number of species present within the BCR sampled, is affected by the time of year, location and habitat. To account for variation in the number of species with the time of year, we include day of year as a model covariate and fit a cyclic cubic regression spline with 20 degrees of freedom. To control for spatial location we model differences in the average number of species on observers’ checklists for individual BCRs. For this study we report the results from 6 BCRs from widely separated regions (Fig 1) that contain different avian communities. The number of participants and checklist submission rates varied across BCRs (Table 1).

To account for the effect of local habitats within a BCR on the suite of species present we include covariates that describe the land-cover types in the surrounding area. Each eBird observation location is linked to the 2011 MODIS landcover imagery as described by the global land cover product (MCD12Q1) [22] using the University of Maryland classification scheme [23], which classified each 500m × 500m MODIS pixel as belonging to one of 14 landcover classes. We summarized the landcover data as the proportion of each of the landcover classes within a 3km × 3km (900 hectare) pixel centered at each observation location. Locations with several habitats in close proximity are likely to have higher numbers of species, and we include a habitat heterogeneity covariate using the Gini-Simpson diversity index [24,25] applied to the landcover class proportions. The Gini-Simpson diversity index is zero if the 3km × 3km pixel is comprised of one landcover class, to one, for equal proportions for all 14 landcover classes.

### Covariates Describing Differences in the Observation Process

The second covariate category of covariates describe those extrinsic factors that accounted for any differences in an observer’ ability to detect species. Given that a certain number of species was present, the time of day is an important descriptor of species detectability, as bird behavior often varies with time of day with many species being more active and vocal in the morning. We modeled time of day as a regression spline with 5 degrees of freedom; a cyclic spline was not needed, as only checklists from counts that started between 5 am and 8 am were included. We also included effort covariates: distance travelled, the number of observers, and amount of time birding. An observer who travelled further will cover a greater area and therefore likely encounter a greater number of species. While a small group of observers is also likely to record a greater number of species, a large group could interfere with finding a species. To describe the accumulation of species with time, we included the amount of time birding for each checklist and the square root of the amount of time birding in order to allow the model to describe a saturation effect.

### Covariates Describing Observer Variation in Detecting Species

The third category of covariates allowed us to describe the inter- and intra-observer variation in ability to detect species. First, we included as a fixed effect the average variation in species detected across all observers, which accounted for inter-observer variation. To account for intra-observer variation, we fit observer-specific random effects both for the model intercept and the coefficient of the number of hours surveyed. The use of both this random intercept and random slope allowed for inter-observation variation in both the baseline number of species that could be identified by each observer (random intercept), and the rate at which new species were located and identified (random slope). We also tested a random effect of observer on the coefficient of the square root of the number of hours, but this did not result in model improvement and was not included. To test for changes in observers’ abilities with increased experience, checklists were indexed in the order in which they were submitted to eBird. An observer’s first checklist submitted between 2004–2012 had an index of one and the index increased chronologically thereafter. The logarithm of the checklist index was included as a covariate in the model. This covariate allowed for participant’s rates of species accumulation to change (and we presumed increase) with increased time spent birding, and the use of a logarithm allowed for the rate of increase to plateau.

### The Species Accumulation Curve Index

The Poisson generalized additive mixed model used in our analysis had the following form:

$$\text{log}\left(N_{k}\right)=\;\alpha_{0}+\;\alpha_{1}hrs_{k}+\alpha_{2}\sqrt{hrs_{k}}+\overset{14}{\underset{j=1}{\sum }}\beta_{j}land_{j,k}+\beta_{15}GS_{k}+f_{1}\left(day_{k}\right)+\gamma_{1}protocol_{k}+\gamma_{2}dist.km_{k}+\gamma_{3}no.observers_{k}+f_{2}\left(time_{k}\right)+\delta_{1i}+\delta_{2i}hrs_{k}+\lambda_{1}\text{log}\left(checklist.no_{i,k}\right)+\varepsilon_{k}$$

$$\delta_{1i}\sim N\left(0,\sigma_{1}\right)\delta_{2i}\sim N\left(0,\sigma_{2}\right)$$

Here the variable *k* denotes a checklist and *i* denotes the observer who submitted checklist *k*. *N*_*k*_ is the number of species observed on checklist k and *hrs*_*k*_ is the number of hours spent birding on checklist *k*; *land*_*j*,*k*_ is the proportion of land cover for category *j* and *GS*_*k*_ is the Gini-Simpson habitat diversity index for the habitat in the 3km × 3km area centered on checklist *k; day*_*k*_ is the day of the year and *f*_*1*_ the cyclic smooth; *protocol*_*k*_ describes whether the observer of checklist *k* stood in one location or traveled and *dist*.*km*_*k*_ is the distance travelled; *no*.*observers*_*k*_ is the number of observers recorded as part of the group for checklist *k*; *time*_*k*_ is the time of day checklist *k* began and *f*_*2*_ is a thin plate regression spline; *checklist*.*no*_*k*,*i*_ is the sequential number of checklist *k* within all the checklists submitted by observer *i*.

The first row of this formula and the α coefficients describe the increase in species observed with more time spent in collecting data for each observation: the basic SAC. The second row and the β coefficients describe the covariates that affect the number of species present. The third row and the γ coefficients describe the availability of species for detection and the effort expended for a given checklist. The δ coefficients are random variables that describe the observer-specific effects for the intercept and the slope of the SAC. These are distributed according to a normal distribution, and initial testing with unconstrained (i.e., fixed) observer effects found this to be a reasonable assumption. The λ coefficient describes the average improvement in the rate of detection of species, across all observers, with increased numbers of checklists submitted.

Sample sizes within each of the six BCRs ranged from 72,572 to 312,987 checklists with a mean of 137,618 checklists (Table 1). We had strong *a priori* expectations that all of the covariates included would have an impact on the number of species observed. As a result model selection was not carried out, as the purpose of including all the covariates was to describe as much of the variation as possible and obtain the most accurate estimates of observer ability to detect and identify species of birds. Models were run in R version 3.1.3 [26] with packages ‘mgcv’ [27] and ‘nlme’ [28] to implement the GAMM.

### Individual Species Accumulation Curve Indices

To quantify an individual observer’s proficiency for finding and identifying bird species, we used the SAC models to predict the expected number of species reported by each observer, had all observers conducted the same standardized search. The standardization was done to control for the modeled factors extrinsic to skill. We refer to this value as an observer’s SAC index. Specifically, for each observer in a BCR we used the models to predict the expected number of species that each observer would report from a one-hour search starting at 7am, travelling 1km on the 1^st^ of September. For predictions, landcover covariates for each BCR were set at their mean values and then standardized to sum to 100%. Habitat diversity was calculated for these standardized mean values. The number of checklists was set to 100 for all observers in calculating their SAC indices. Alternative calculations of indices, setting observation times to greater or less than 1 hour were very highly correlated with the 1-hour scores, indicating that the index was not sensitive to the choice of 1 hour. We chose to standardize the index for the 1^st^ of September because there is a relatively large number of species available for detection across the United States at that time of year, providing a strong signal to discriminate observer differences. Moreover, we believe the largest component of observers’ ability to detect and identify species will not vary substantially throughout the year, providing justification for the use of a score standardized for this specific date.

### Comparing Species Accumulation Curve Indices

To understand the causes of inter-observer variation in SAC indices, we ranked observers from lowest to highest SAC index, and we compared the detection rates of individual species from observers in the lowest quartile of the SAC index distribution and observers in the highest quartile of the SAC index distribution within each BCR. For each species, we calculated the proportion of checklists in which a species occurred for all checklists submitted by observers in the lower quartile group and separately for all checklists by observers in the upper quartile. We included only species seen on at least 1% of the checklists, as particularly rare species will not present a good comparison of detection rates between the two groups of observers. Resampling the data within each group of observers, using the observer as the unit of bootstrap resampling produced bootstrap confidence intervals for the detection rates. Two hundred bootstrap samples were produced for the each group and 95% confidence intervals were calculated for the detection rates. We plotted barplots of the detection rates for both groups of observers for the 20 species that had most similar detection rates across the two groups and the 20 species that had the most different detection rates across the two groups. We made this comparison in order to determine whether all species, or only rare species, are detected more readily by more experienced observers.

We used two validation methods to compare detection rates of species conditioned on the two observer groups with SAC indices from the lowest and highest groups. The first qualitative method had experts at the Cornell Lab of Ornithology in bird identification and geographic occurrence interpret the reasons for some species being detected at similar rates by the two groups of observers. The second quantitative method was carried out to investigate if the detection rates between the two groups are consistent across all species. An alternative hypothesis is that the difference between the detection rates in the two groups is greater for species that are detected less often. As species become more common, the detection rates for the lower quartile group would therefore get closer to the detection rates for the upper quartile group. Detection rates were compared across all species and the upper quartile group’s detection rate was modeled as a function of the lower quartile group’s detection rate. Both detection rates were logit-transformed and the square of the logit novice detection rate was also included in the model so that a quadratic model was fitted to these data.

### Comparing an individual’s SAC Index over time

To assess whether observers improved their species accumulation rate over time we included the λ coefficient in our model to describe the average learning rate of participants. A birder's first checklist between 2004–2012 has a checklist index of one and the index increases sequentially thereafter. This variable gives us an indication of a birder's experience with eBird, and allows us to look at the longitudinal participation of birders to see how the number of species they detect changes as they submit more checklists.

### Improving Citizen Science Data Quality

To assess if individual observer’s scores can be used to control for observer differences when analyzing eBird data, we tested the impact of including the scores in species distribution models. *A priori*, we expected that including a covariate that effectively captures variation in individual observer ability would improve the predictive performance of distribution models that otherwise lack any information about observer score. Additionally, we expected that the difference in predictive performance would tend to be greater for species that are hard to detect, because observer ability will be a more important source of variation in species detection. To test these ideas, we ran models for 10 species which are easy to detect and identify, the species for which the lowest quartile detection rates were closest to those of highest quartile, and 10 species which require significant abilities to detect or identify, the species for which lowest quartile detection rates were the most different to those of highest quartile. Separate models were run for 20 species within each of the six BCRs.

The species distribution models included the presence of a species on a checklist as the response variable with a binomial error structure and a logit link function. Algebraically, the model is:

$$\text{logit}\left(P_{k}\right)=\alpha_{0}+\alpha_{1}hrs_{k}+\alpha_{2}protocol_{k}+\alpha_{3}dist.km_{k}+\alpha_{4}no.observers_{k}+f_{1}\left(time_{k}\right)+f_{2}\left(skill_{i}\right)+\overset{14}{\underset{j=1}{\sum }}\beta_{j}land_{j,k}+\beta_{15}latitude+\beta_{16}longitude+f_{3}\left(day_{k}\right)$$

The variable *k* denotes a checklist and *i* denotes the observer who submitted checklist *k*. *P*_*k*_ is a binary variable denoting the presence or absence of a species on checklist *k*; *protocol*_*k*_ describes whether the observer of checklist *k* stood in one location or travelled and *dist*.*km*_*k*_ is the distance travelled; *no*.*observers*_*k*_ is the number of observers recorded as part of the group for checklist *k*; *time*_*k*_ is the time of day checklist *k* began and *f*_*1*_ is a thin plate regression spline; *skill*_*i*_ is the data submission score for observer *i* and *f*_*2*_ is a thin plate regression spline; *land*_*j*,*k*_ is the proportion of land-cover for category *j*; *day*_*k*_ is the day of the year and *f*_*3*_ the cyclic smooth. The *α* coefficients describe the effect of covariates describing effort or detectability. The *β* coefficients describe the effect of location and habitat covariates.

To assess the impact of including the data submission score as a covariate, models were run for each species with and without the score smooth. Model selection was not carried out, as the purpose of including all covariates was to describe as much of the variation as possible and ascertain whether the scores led to improvement in the complete model. Models were run in R [26] with package ‘mgcv’ [29].

The models were run with a training dataset, which comprised 90% of the checklists in each BCR. Performance of the species distribution models was assessed on the remaining 10% of the data. The performance of the two distribution models for each species were assessed with two performance metrics commonly used for species distribution models: Area Under the Curve (AUC) and Kappa [30–32]. For each species, Kappa and AUC are calculated for the model without the data submission score and the model with the data submission score effect. The differences in performance metrics from the two models were compared for each species.

## Results

For the sake of brevity, we provide results from two of the six BCRs that were analyzed. The results for the four other BCRs are provided in the Supporting Information. We selected BCR 23—Prairie Hardwood Transition and BCR 31—Peninsular Florida (Fig 1) as our representative examples. These two BCRs were selected for their unique differences: BCR 31 had many participants and experiences dramatic seasonal changes in the species composition, while BCR 23 had fewer participants and lower seasonal changes in species composition.

### Observer Differences in Species Accumulation Curves

**Fig 2:**
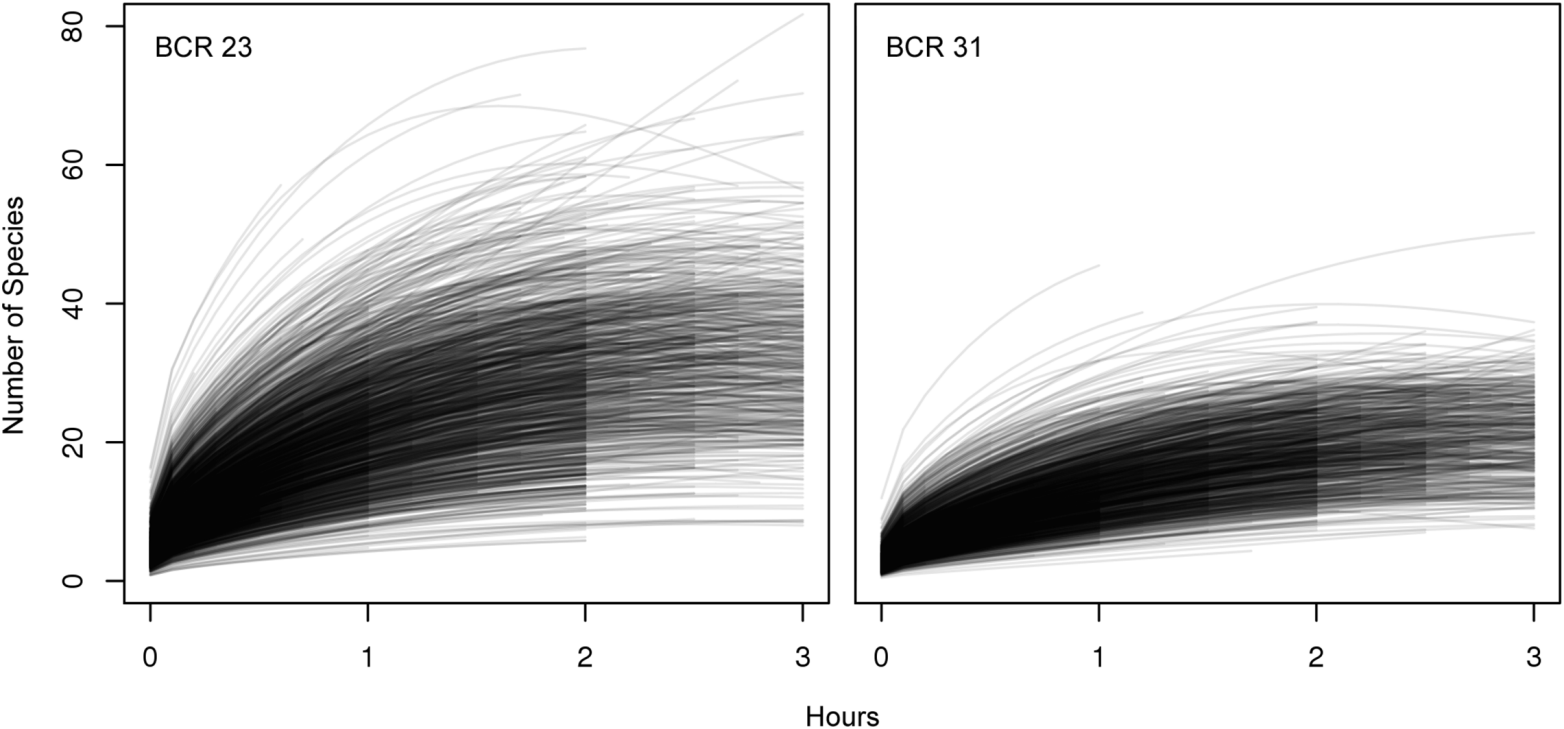
Species accumulation curves for all individual observers in a BCR. Each line represents the species accumulation curve derived from the mixed model fit to data from that BCR, for every individual observer in that BCR, and calculated with the standardized covariates of Sep 1^st^, 7am start time, travelling 1km and average percentage land cover. The fitted line for each observer is plotted to the maximum count period duration in the data from that observer. Species accumulation curves that decrease for some observers may indicate different biases in attention to birding. For example checklists under 1 hour may be more concentrated birding, whereas checklists over 1 hour may combine birding with another activity such as hiking or fishing.

**Fig 3:**
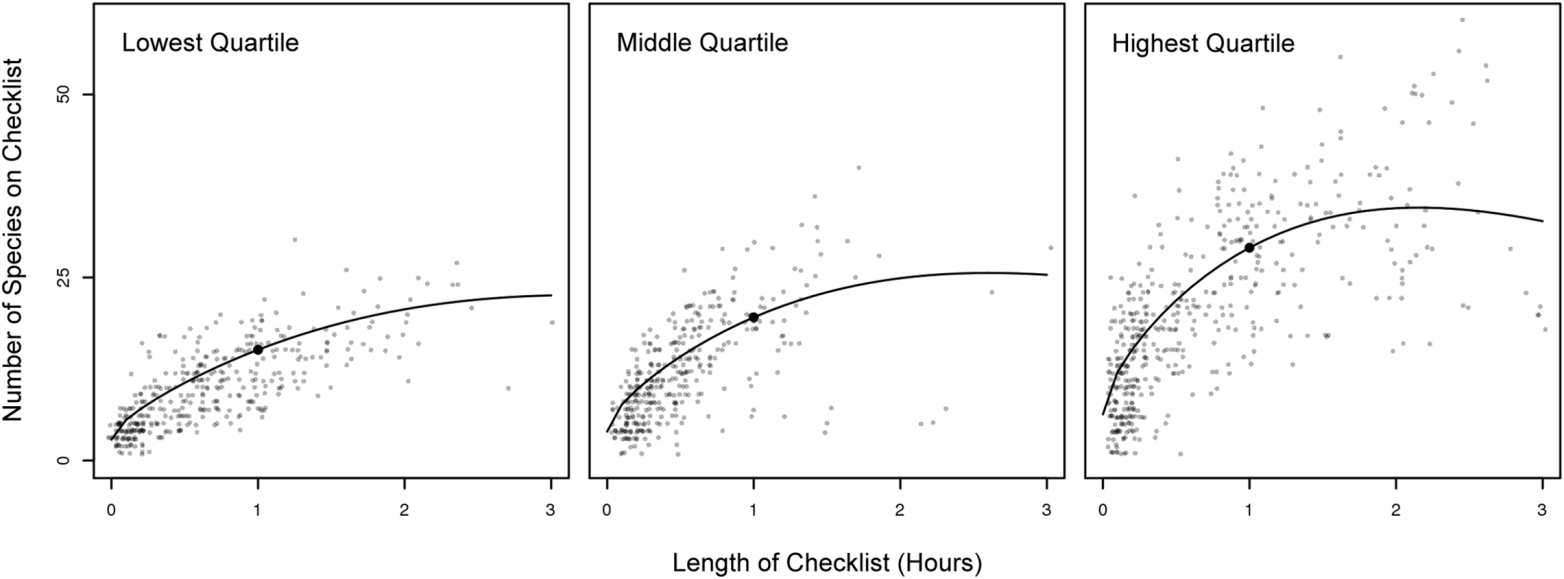
Representative Individual Species Accumulation Curves and Indices. Actual counts of species reported and observer-specific SACs for one individual observer classified as within the lowest quartile a), middle quartiles b), and highest quartile c). As the duration an observer spends collecting data for each checklist increases, the number of species observed increases. However as the duration lengthens, the rate of species accumulation decreases. The black dot on each curve is the SAC Index—the estimated number of species that individual would see during one hour of birding. Individuals were selected from the group who submitted at least 300 checklists between 2002 and 2012.

SACs provided a good measure of inter-observer variability in eBird participants. We found high observer variation in SACs (Fig 2, S1 and S2 Figs). We attribute the greater variation in individual SACs from BCR 23 to more participants, more species available, and higher difficulty in making species identifications of migrant birds (see section on Qualitative Differences in Species Reported). When SACs were viewed for individual observers we found that as expected, the number of bird species reported initially increased rapidly with longer periods of observation but when durations exceeded one hour the increase number of species reported began to approach an asymptote (Fig 3).

### Observer Differences in Species Reported

**Fig 4:**
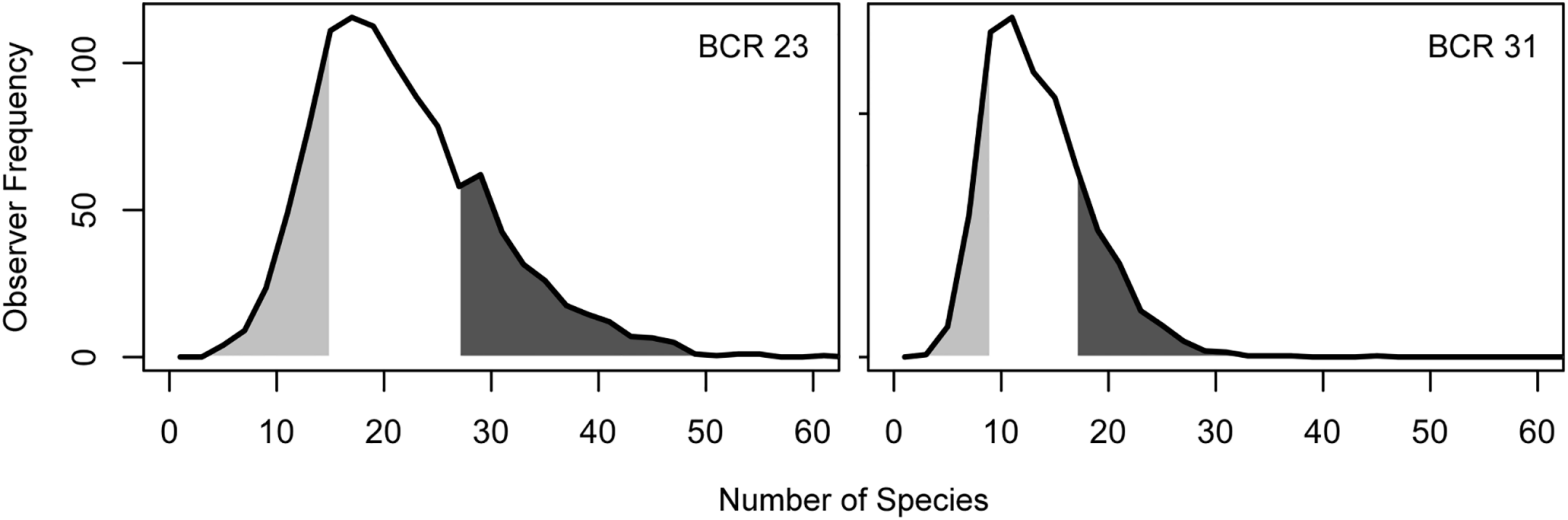
Distribution of individual SAC Indices. The expected number of species observed in 1 hour for all observers in a BCR. Individual data submission scores are ranked from lowest to highest and the light gray region represents the lower quartile of observers, and the dark gray region the upper quartile of observers.

**Fig 5:**
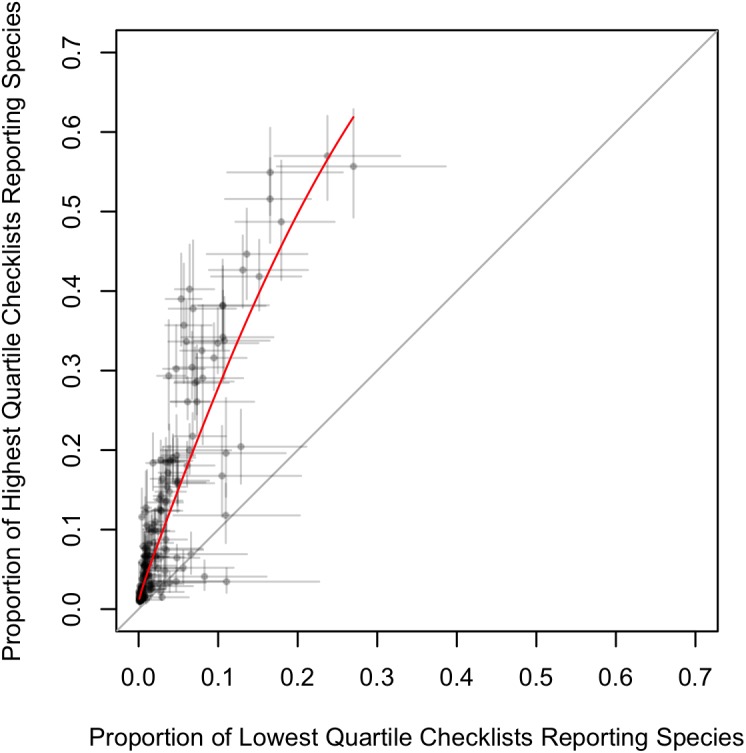
Comparison of detection rates of individual species of birds for observers in the lower quartile and the upper quartiles of SAC indices. Detection rates are the proportions of checklists that record a given species and lines represent 95% bootstrap confidence intervals. The red line is a model fitted to the logit detection rates. The gray line indicates the line of equality, where detection rates for the two groups are equal; the highest quartile had statistically significant detection rates for the majority of species.

**Fig 6:**
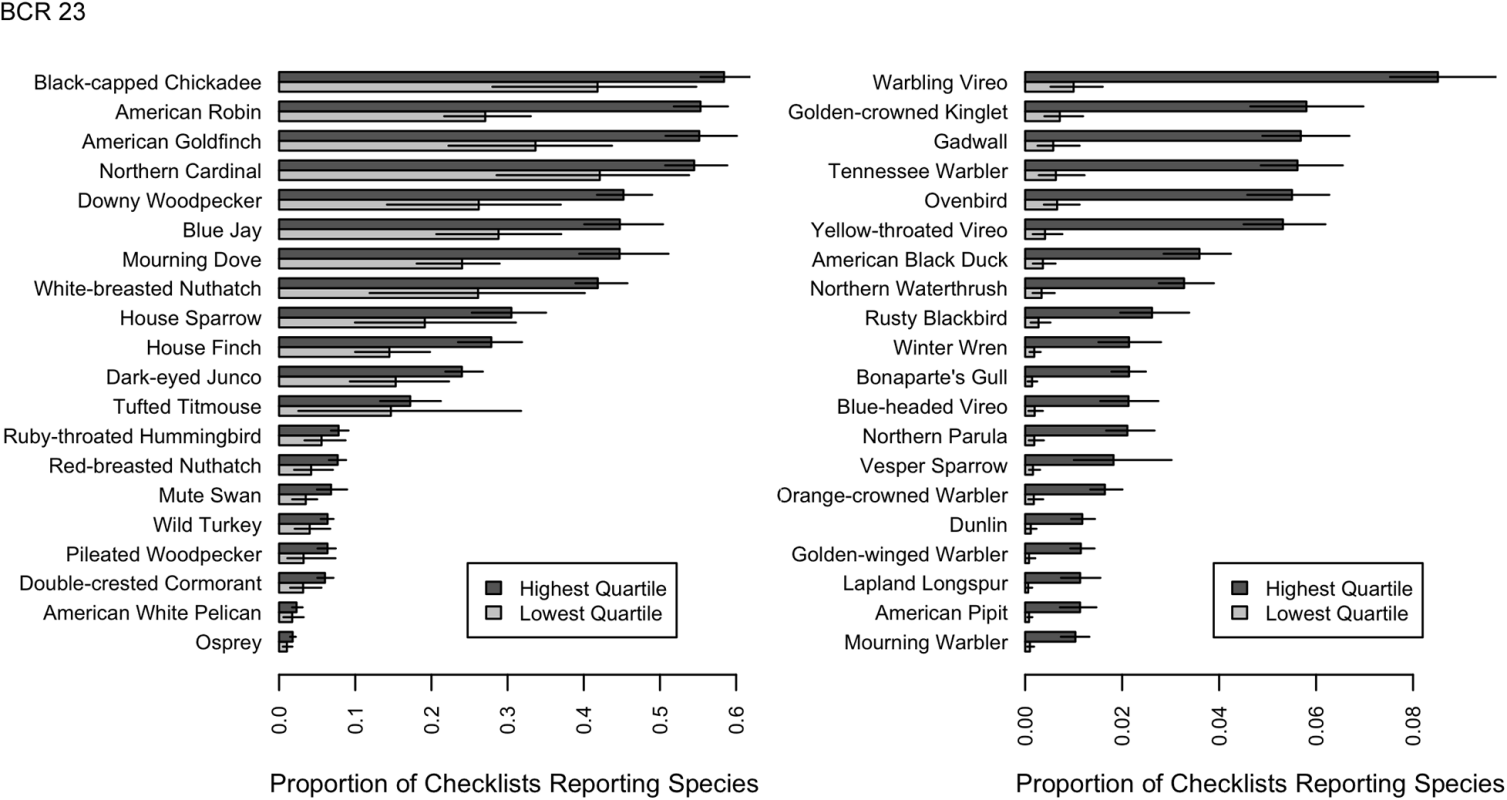
Bird species detected at the most similar and dis-similar rates by observers in the lowest quartile and highest quartile of SAC index values. Barplots from BCR 23 of the 20 species for which detection rates are proportionally most similar (left-hand panels) and the 20 species for which detection rates are proportionally most different (right-hand panels). Detection rate is the proportion of checklists that record a given species and error bars represent 95% bootstrap confidence intervals. The 20 species for which the two groups have proportionally most similar detection rates are generally species that are fairly easy to identify by sight. The 20 species that the two groups have proportionally most different detection rates are generally species that are difficult to identify, easier to identify by sound, or often be seen as a high-flying silhouette without many distinguishing features.

**Fig 7:**
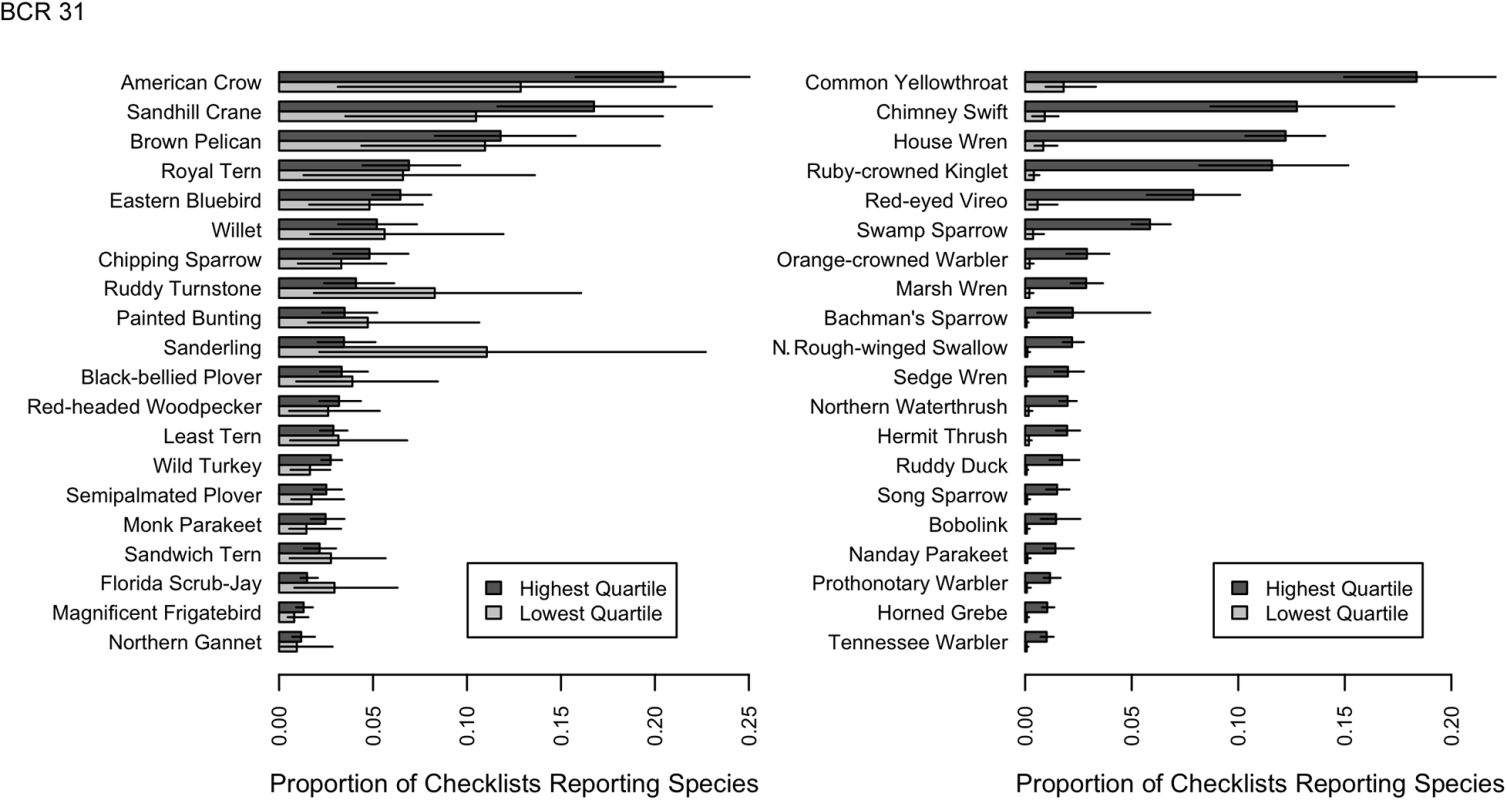
Bird species detected at the most similar and dis-similar rates by observers in the lowest quartile and highest quartile of SAC index values. Barplots from BCR 31 of the 20 species for which detection rates are proportionally most similar (left-hand panels) and the 20 species for which detection rates are proportionally most different (right-hand panels). Detection rate is the proportion of checklists that record a given species and error bars represent 95% bootstrap confidence intervals. The 20 species for which the two groups have proportionally most similar detection rates are generally species that are fairly easy to identify by sight. The 20 species that the two groups have proportionally most different detection rates are generally species that are difficult to identify, easier to identify by sound, or often be seen as a high-flying silhouette without many distinguishing features.

We found that expertise is not just the ability to detect more rare species, but a higher rate of detection of all species. To explore patterns with the inter-observer variation we grouped all observers by ranking individual data submission scores from lowest to highest and then grouped participants in the lowest and highest quartile scores for further analysis (Fig 4, S3 and S4 Figs). Individuals from the highest quartile had the fastest rate of accumulation of species (Fig 3), reported all species with greater frequency (Fig 5) and reported less common species with a much greater frequency (Figs 6 and 7, S5, S6, S7 and S8 Figs).

Individual observers in the highest quartile consistently submitted more checklists (Table 2), which suggests that an individual’s ability and rate of participation are linked. The extreme differences between the mean and median number of checklists submitted indicate that for each group there are a few eBird participants that submit a much higher number of checklists than the typical observer. Furthermore, the greater difference between mean and median for the higher quartile group suggests a greater propensity for these observers to submit more checklists.

**Table 2:** The mean and median number of checklists submitted for the lowest quartile and highest quartile of eBird participants.

BCR	Low Quartile Mean Number of Checklists	High Quartile Mean Number of Checklists	Low Quartile Median Number of Checklists	High Quartile Median Number of Checklists
**9**	**20**	**70**	**4**	**5**
**23**	**21**	**108**	**2**	**4**
**30**	**36**	**126**	**3**	**8**
**31**	**22**	**77**	**3**	**5**
**32**	**14**	**168**	**3**	**6**
**37**	**15**	**49**	**5**	**9**

If the individual SAC Indices effectively discriminate between observers of different skill levels, we would *a priori* expect the observers in the highest quartile to: 1) detect all species at higher rates (Fig 5); 2) are better at identification of species by sound, as this is a harder skill to master (Figs 6 and 7); and 3) are good at detecting secretive and hard-to-identify species, which require experience to locate, detect, and identify with confidence (Figs 6 and 7). To test these hypotheses we compared the rates at which individual species were reported on checklists from observers with SAC indices in the lower quartile with those from the highest quartile. Fig 6 shows the 20 species with the most similar detection rates across the two groups of observers, and the 20 species with the most different detection rates. The species for which there was little difference between reporting rates of the two observer groups (Fig 6) were the species that were highly distinctive and conspicuous and that could be easily detected visually (e.g., Wild Turkey (*Meleagris gallopavo*), Great Blue Heron (*Ardea herodias*), or species that commonly frequent bird feeding stations (e.g., White-breasted Nuthatch (*Sitta carolinensis*), Blue Jay (*Cyanocitta cristata*). By contrast, the species with the greatest disparity in detection rates between the two observer groups (Fig 7) were those that were hard to identify (e.g., gulls), best identified by sound (e.g., flycatchers), secretive in their habits and detected more often by sound (e.g., marshbirds), or present only in very specific habitats that birders proactively seek (e.g., sandpipers and other shorebirds).

As a final comparison, we looked at individual participants within each region. In all cases, people known to be experts (i.e. authors of identification articles, regional editors for birding publications, members of regional review teams) had high SAC indices that always fell within the highest quartile. The eBird Project Leaders, or eBird editors within each region generally did not know individuals within the lower quartile.

### The Impact of Continued Data Submission

**Fig 8:**
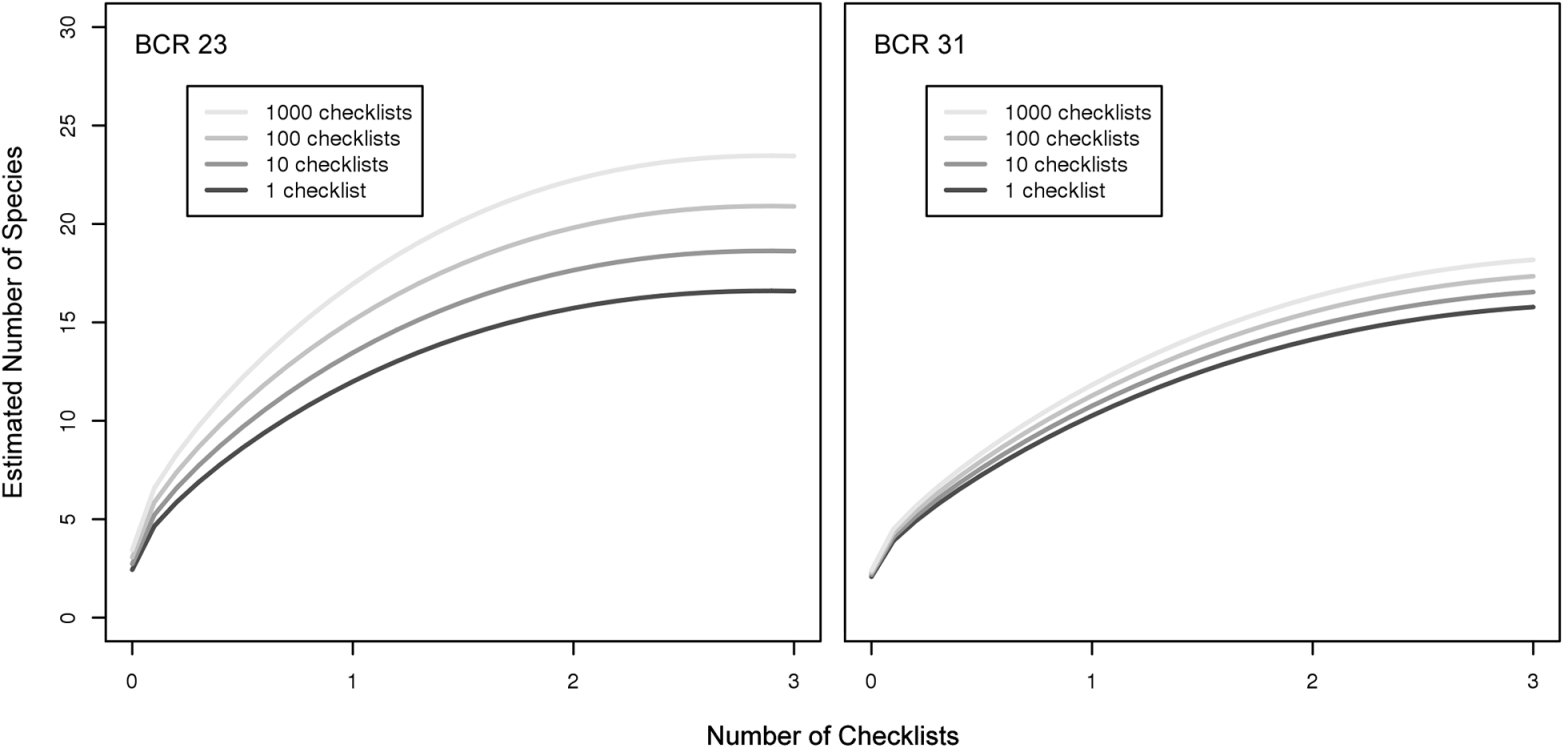
The change in SACs as a function of the cumulative participation in eBird. We estimated average changes in shapes of species accumulation curves with increasing number of checklists submitted to eBird from our BCR-specific models of species accumulation curves, to visualize whether observers report more species after they have submitted more eBird checklists. Note that while increased participation leads to a higher rate of accumulation of species, this effect is highest for beginning participants and slows with increased participation.

The previous results indicate that the observer’s SAC index characterized important, observer differences at detecting and identifying species. By extension, we would *a priori* expect that an observer’s ability in detecting and identifying birds should increase with practice and experience, and therefore eBird participants should accumulate species at higher rates if they spend more time birding, as indicated by the number of checklists submitted to eBird. We found this result within all of the six selected BCRs (Fig 8, S9 and S10 Figs). Additionally, the rate of improvement slowed with increased participation, where the expected improvement between the first and 100^th^ checklists was the same as the improvement between the 100^th^ and 1000^th^ checklists. The diminishing returns observed for the rate of increase follows from the fact that the logarithm of checklist number was used as a covariate. In preliminary analyses we tried several transformations (i.e., linear checklist number and square root of checklist number) and found that the logarithm of checklist number produced the most statistically significant results. While we found that species accumulation curves improve, and that improvement decreases with increased participation across all BCRs, the number of species in a BCR compounded the magnitude of the differences. Our model indicates that participants in BCRs with more species (i.e., BCR 23) showed greater improvement than participants in BCRs with fewer species (BCR 31).

### The Impact of including SAC Indices in Species Distribution Models

**Fig 9:**
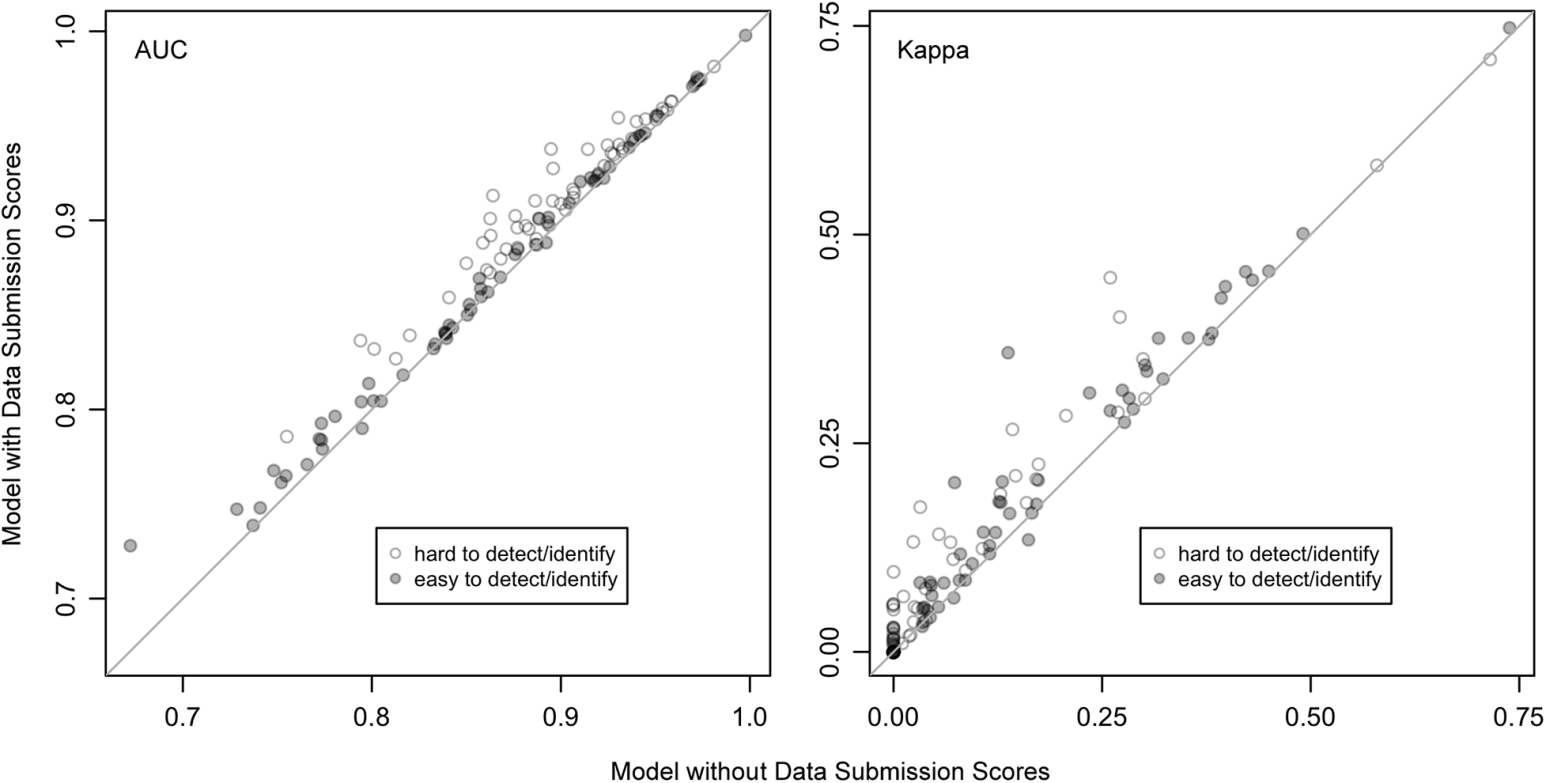
Species distribution model accuracy with and without data submission scores (AUC a and Kappa b.). There are 20 species models from each of the 6 BCRs: 10 for species, which are hard to identify, and 10 for species that are easier to identify, as defined by differences between the highest and lowest quartiles of data submission scores (Figs 6 and 7). In most cases the inclusion individual observer data submission scores improved model accuracy.

The addition of the data submission scores in the model led to improved model performance for 93% (AUC) and 88% (Kappa) of the 120 species distribution models fit across the 6 BCRs (Fig 9). In the few cases where the addition of the expertise covariate led to a reduction in model performance, this decrease was small.

## Discussion

Data are always descriptions of reality that have been filtered and interpreted through the process of being recorded, regardless of quality of the recording process. In quantifying observer rates at species accumulation over the course of a bout of birding (i.e., collecting data for an eBird checklist), we are dealing with inter-observer variation as a calibration problem. By examining data coming from observers with a range of abilities, we have been able to characterize important axes along which proficiency differs, show that observers improve with practice, and knowledge of inter-observer variation improves data analyses.

As is typical with projects that rely on public participation, a small subset of eBird participants contribute most of the observations [33]. We show that on average these participants contribute higher quality data lessening the trade-off between data quality and quantity. Moreover, the SAC index offers an objective quantitative measure that can be used to identify and rank data quality based on observer differences. Thus, used in other analyses the SAC index provides a mechanism to account for and control an important source of bias in eBird. More generally, we suggest that the potential to derive post-hoc data-derived measurements of participant ability should be more widely explored by analysts of data from citizen science projects. We see the potential for inferential results from analyses of citizen science data to be improved by accounting for individual differences in observers.

We recognize that while this study addresses only one aspect of data quality in citizen science projects, there are additional issues that remain to be addressed such as the irregular spatial distribution of observations [7]. However, our finding that the act of continued participation leads to improved ability suggests that there is general value to create mechanisms in citizen-science projects that encourage continued project participation.

Our results show that the quality of data submission by participants in eBird on average improves in a systematic fashion over time through increased practice as indexed by the number of checklists submitted by each observer. While many citizen science projects presume that such learning occurs, few projects have attempted to make direct measurements of this learning process [34,35]. We suggest that while eBird provides the incentive to continue birding, it is the act of birding that improves an individual’s observational skills. The more an observer goes birding the more familiar they become with the avifauna in their region, which increases their ability to detect, identify, and finally to report more species to eBird. Birding is a highly nuanced cognitive activity that involves using visual and auditory cues to make identifications. This combination of both sensory and cognitive abilities improves as the observer puts more effort in the act of birding. As participants learn to become better observers they improve the desired scientific outcomes of the eBird project as a whole, which is exemplified by the improvement in eBird species distribution models.

In summary, the dual goals of citizen science projects—participation and learning, and collection of valid data for scientific research—are actually closely linked because as participants become more familiar with the goals of the project, the quality of the data they submit improves.

### Data Quality Implications for Citizen Science

Concerns about the quality of data collected in citizen science projects most often focus on concerns over false positives—the erroneous reporting of species that are not present. Successful citizen science projects have data quality measures in place to minimize the volume of false positives. The methods employed by eBird uses expert-developed filters to flag observations that are unexpected for a given place or time [17,19]. Volunteer experts in species distributions then review the merits of each record individually, including photographic documentation when available, followed by communications with the original observers when necessary. Comparable methodology of expert review has been used in other citizen science projects such as eButterfly (http://e-butterfly.org), iNaturalist (www.inaturalist.org) and REEF (www.reef.org) and is generally accepted as a method to reduce false positives to an acceptable level.

However, false negatives—failing to report a species that is present—are much harder for citizen science projects to address. When common but hard-to-detect species are systematically underreported (Figs 6 and 7), analyses based upon those data will also underrepresent that species true occurrence or abundance. While occupancy modeling [36] accounts for variation in detection rates, it cannot distinguish the detection rates among individuals observers without information about those individuals. The SAC index is a good univariate source for this information.

Our methodology assigns a SAC index that effectively measures differences in observers’ false negative error rate. Observers with high SAC indices are detecting a high proportion of the species available for detection and thus have low false negative error rates. Conversely, observers with comparatively low SAC indices are missing a large number of species that are available for detection and thus have comparatively high rates of false negatives. It is likely that false positive and false negative error rates co-vary, and the incorporation of SAC indices in existing data analyses will prove beneficial in improving the inferences that can be made from citizen science data.

We are not aware of any published cases in which the effects of learning by participants have been accounted for in a quantitative way during analysis. Instead, studies have evaluated volunteer performance in citizen science projects [37] through ground truthing [38], comparing their observations with those provided by experts [39], or incorporated observer identity as a covariate [40] with the implicit assumption that proficiency can be treated as a static measure.

In this paper, we have shown that observers accumulate species at different rates, the rate of accumulation increases with continued participation, and the species that are missed from observers in the lowest quartile of SAC indices are a consistent set of species that are hard to identify or require specific knowledge of the habitats they occur. We conclude that these patterns relate to the underlying, but impossible to measure, latent attribute of skill. In future work, we would like to explore the extensibility of the data quality mechanisms that we have described as the approach to indexing observer ability should apply to numerous biodiversity-monitoring projects that are established in most countries globally, both with data collected by trained observers [41–44] as well as data from citizen science projects.

## Supporting Information

S1 Fig BCR 32 and BCR 37—Species accumulation curves for individual observers. Each line represents the species accumulation curve for a single observer, calculated with the standardized covariates of Sep 1^st^, 7am, travelling 1km and average percentage land cover. The fitted line for each observer is plotted to the maximum checklist length for that observer. Species accumulation curves that decrease for some observers may indicate different biases in attention to birding. For example checklists under 1 hour may be more concentrated birding, whereas checklists over 1 hour may combine birding with another activity such as hiking or fishing.
(TIF)

S2 Fig BCR 32 and BCR 37—Species accumulation curves for individual observers. Each line represents the species accumulation curve for a single observer, calculated with the standardized covariates of Sep 1^st^, 7am, travelling 1km and average percentage land cover. The fitted line for each observer is plotted to the maximum checklist length for that observer. Species accumulation curves that decrease for some observers may indicate different biases in attention to birding. For example checklists under 1 hour may be more concentrated birding, whereas checklists over 1 hour may combine birding with another activity such as hiking or fishing.
(TIF)

S3 Fig BCR 9 and BCR 30—Distribution of individual data submission scores. The expected number of species observed in 1 hour for all observers in a BCR. Individual data submission scores are ranked from lowest to highest and the light gray region represents the lower quartile of observers, and the The second quantitative method The second quantitative method region the upper quartile of observers.
(TIF)

S4 Fig BCR 32 and BCR 37—Distribution of individual data submission scores. The expected number of species observed in 1 hour for all observers in a BCR. Individual data submission scores are ranked from lowest to highest and the light gray region represents the lower quartile of observers, and the The second quantitative method The second quantitative method region the upper quartile of observers.
(TIF)

S5 Fig BCR 9—Bird species detected at the most similar and dis-similar rates by observers in the lowest quartile and highest quartile of SAC index values. Barplots of the 20 species for which detection rates are proportionally most similar (left) and the 20 species for which detection rates are proportionally most different (right). Detection rate is the proportion of checklists that record a given species and error bars represent 95% bootstrap confidence intervals. The 20 species for which the two groups have proportionally most similar detection rates are generally species that are fairly easy to identify by sight. The 20 species that the two groups have proportionally most different detection rates are generally species that are difficult to identify, easier to identify by sound, or often be seen as a high-flying silhouette without many distinguishing features.
(TIF)

S6 Fig BCR 30—Bird species detected at the most similar and dis-similar rates by observers in the lowest quartile and highest quartile of SAC index values. Barplots of the 20 species for which detection rates are proportionally most similar (left) and the 20 species for which detection rates are proportionally most different (right). Detection rate is the proportion of checklists that record a given species and error bars represent 95% bootstrap confidence intervals. The 20 species for which the two groups have proportionally most similar detection rates are generally species that are fairly easy to identify by sight. The 20 species that the two groups have proportionally most different detection rates are generally species that are difficult to identify, easier to identify by sound, or often be seen as a high-flying silhouette without many distinguishing features.
(TIF)

S7 Fig BCR 32—Bird species detected at the most similar and dis-similar rates by observers in the lowest quartile and highest quartile of SAC index values. Barplots of the 20 species for which detection rates are proportionally most similar (left) and the 20 species for which detection rates are proportionally most different (right). Detection rate is the proportion of checklists that record a given species and error bars represent 95% bootstrap confidence intervals. The 20 species for which the two groups have proportionally most similar detection rates are generally species that are fairly easy to identify by sight. The 20 species that the two groups have proportionally most different detection rates are generally species that are difficult to identify, easier to identify by sound, or often be seen as a high-flying silhouette without many distinguishing features.
(TIF)

S8 Fig BCR 37—Bird species detected at the most similar and dis-similar rates by observers in the lowest quartile and highest quartile of SAC index values. Barplots of the 20 species for which detection rates are proportionally most similar (left) and the 20 species for which detection rates are proportionally most different (right). Detection rate is the proportion of checklists that record a given species and error bars represent 95% bootstrap confidence intervals. The 20 species for which the two groups have proportionally most similar detection rates are generally species that are fairly easy to identify by sight. The 20 species that the two groups have proportionally most different detection rates are generally species that are difficult to identify, easier to identify by sound, or often be seen as a high-flying silhouette without many distinguishing features.
(TIF)

S9 Fig BCR 9 and BCR 30—The change in SACs as a function of the cumulative participation in eBird. We estimated changes in the number of species observed with increasing number of checklists submitted to eBird, to test whether observers report more species after they have submitted more eBird checklists. To do this we included a covariate of the log of checklist number, which increased sequentially within each observer. Note that while increased participation leads to a higher rate of accumulation of species, this effect is highest for beginning participants and slows with increased participation.
(TIF)

S10 Fig BCR 32 and BCR 37—The change in SACs as a function of the cumulative participation in eBird. We estimated changes in the number of species observed with increasing number of checklists submitted to eBird, to test whether observers report more species after they have submitted more eBird checklists. To do this we included a covariate of the log of checklist number, which increased sequentially within each observer. Note that while increased participation leads to a higher rate of accumulation of species, this effect is highest for beginning participants and slows with increased participation.
(TIF)
