# Supplementary figures and images for: Can Observation Skills of Citizen Scientists Be Estimated Using Species Accumulation Curves?
Source: PLoS One. 2015 Oct 9;10(10):e0139600. doi: 10.1371/journal.pone.0139600 (PMC4599805; doi:10.1371/journal.pone.0139600)

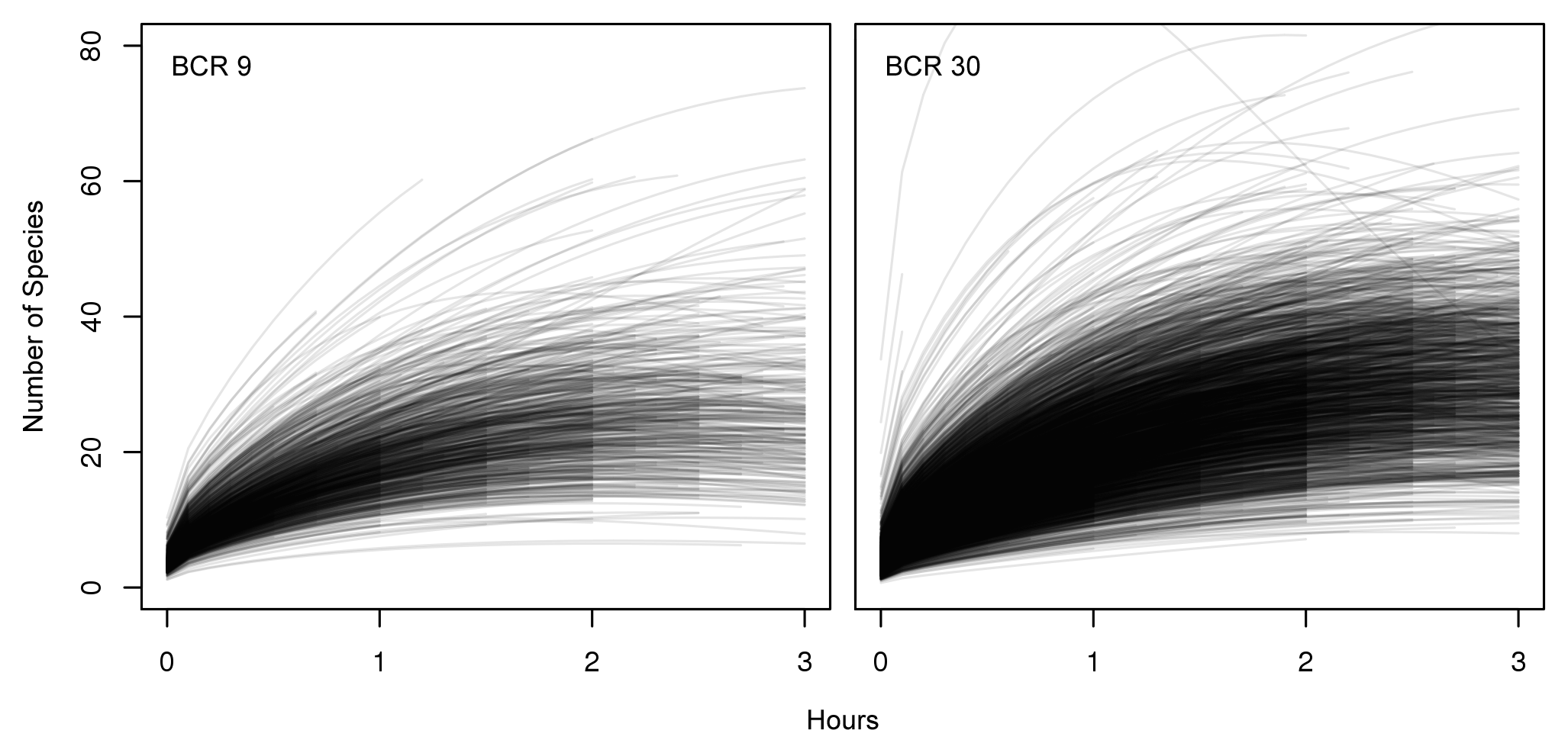

Supplement: S1 Fig — Each line represents the species accumulation curve for a single observer, calculated with the standardized covariates of Sep 1st, 7am, travelling 1km and average percentage land cover. The fitted line for each observer is plotted to the maximum checklist length for that observer. Species accumulation curves that decrease for some observers may indicate different biases in attention to birding. For example checklists under 1 hour may be more concentrated birding, whereas checklists over 1 hour may combine birding with another activity such as hiking or fishing. (TIF) [file pone.0139600.s001.tif]

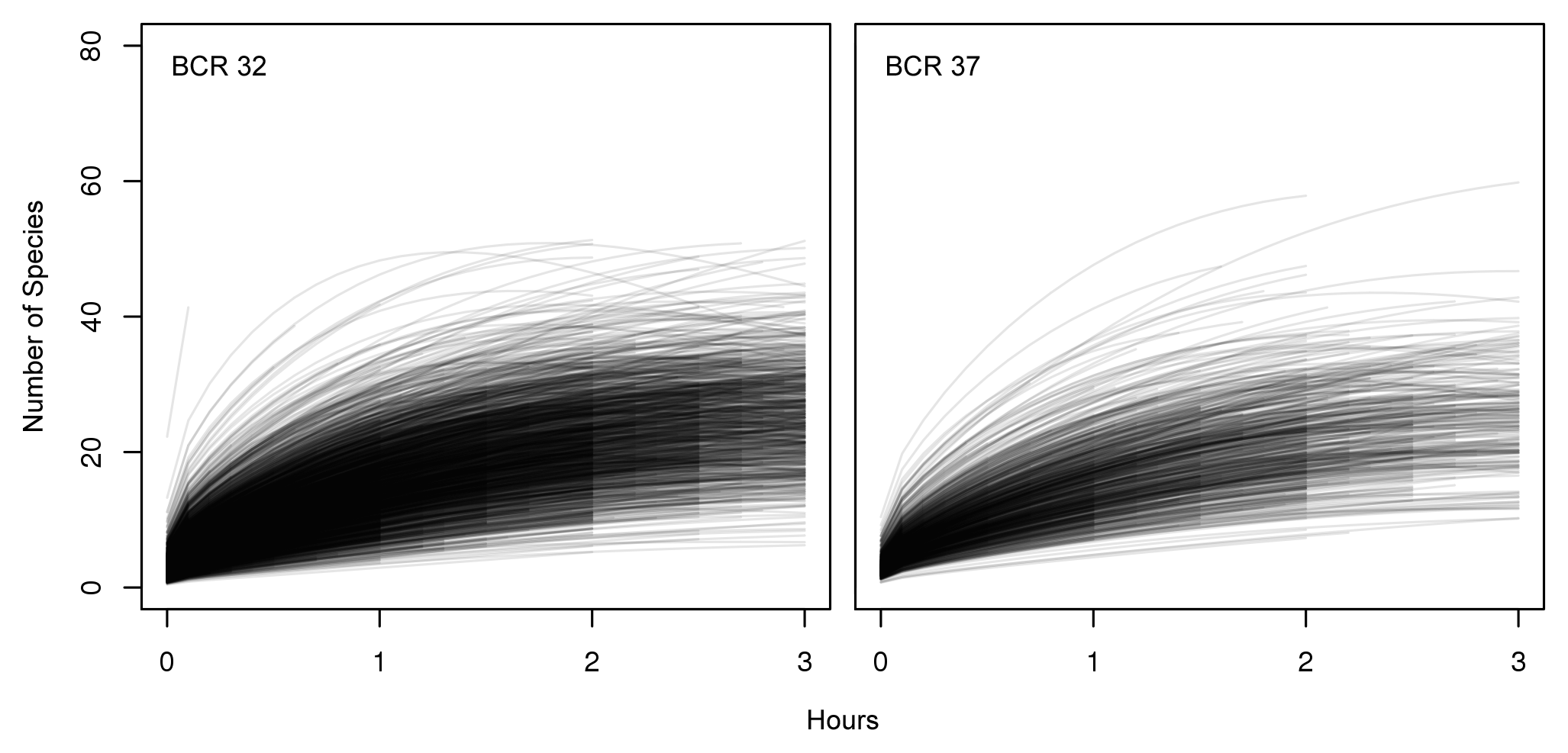

Supplement: S2 Fig — Each line represents the species accumulation curve for a single observer, calculated with the standardized covariates of Sep 1st, 7am, travelling 1km and average percentage land cover. The fitted line for each observer is plotted to the maximum checklist length for that observer. Species accumulation curves that decrease for some observers may indicate different biases in attention to birding. For example checklists under 1 hour may be more concentrated birding, whereas checklists over 1 hour may combine birding with another activity such as hiking or fishing. (TIF) [file pone.0139600.s002.tif]

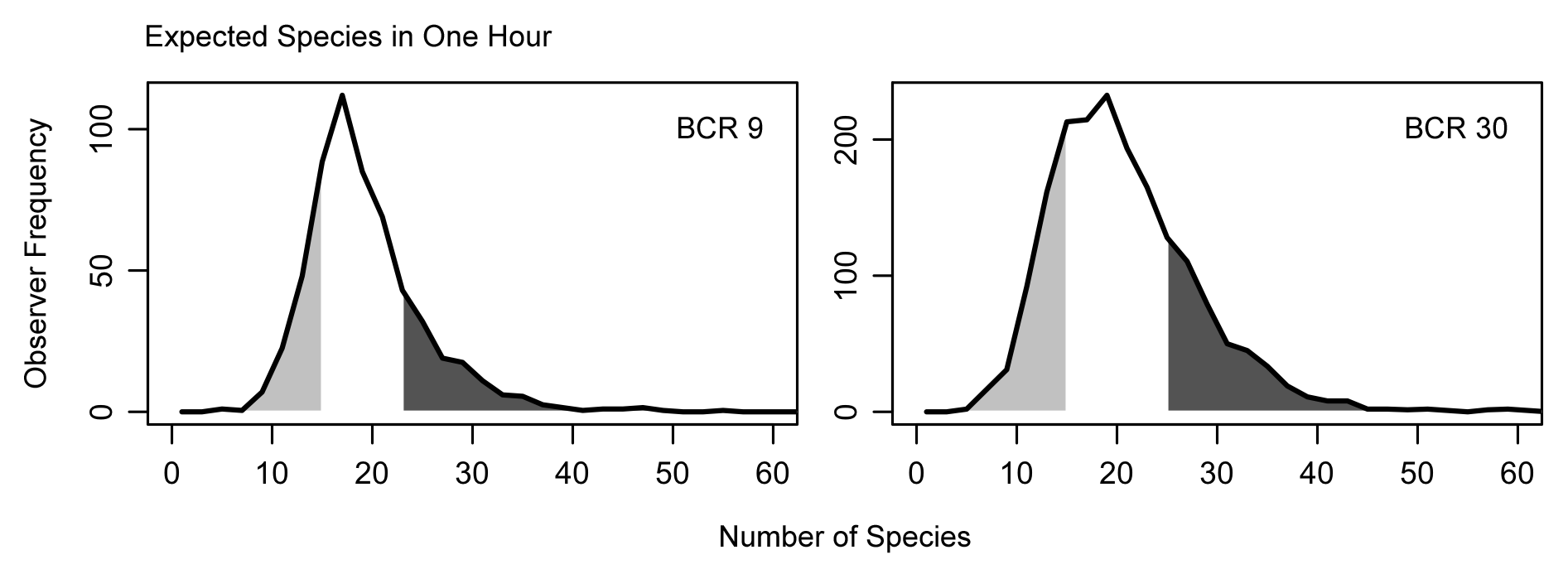

Supplement: S3 Fig — The expected number of species observed in 1 hour for all observers in a BCR. Individual data submission scores are ranked from lowest to highest and the light gray region represents the lower quartile of observers, and the The second quantitative method The second quantitative method region the upper quartile of observers. (TIF) [file pone.0139600.s003.tif]

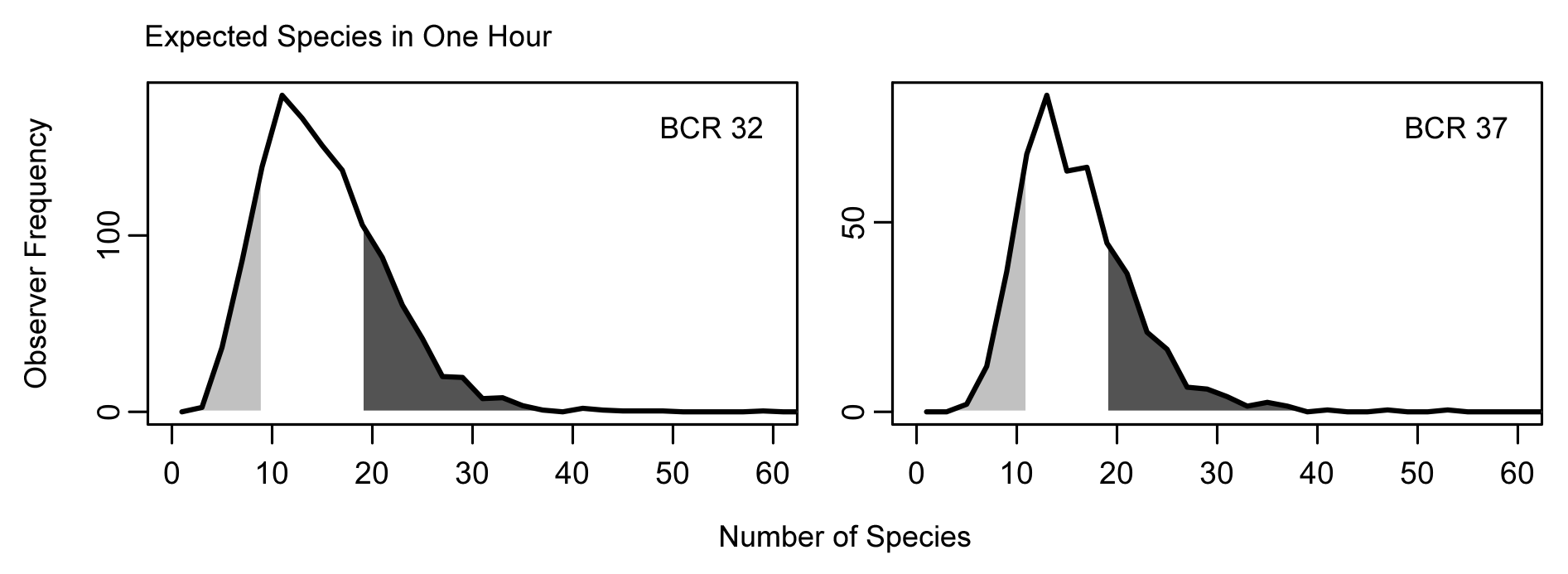

Supplement: S4 Fig — The expected number of species observed in 1 hour for all observers in a BCR. Individual data submission scores are ranked from lowest to highest and the light gray region represents the lower quartile of observers, and the The second quantitative method The second quantitative method region the upper quartile of observers. (TIF) [file pone.0139600.s004.tif]

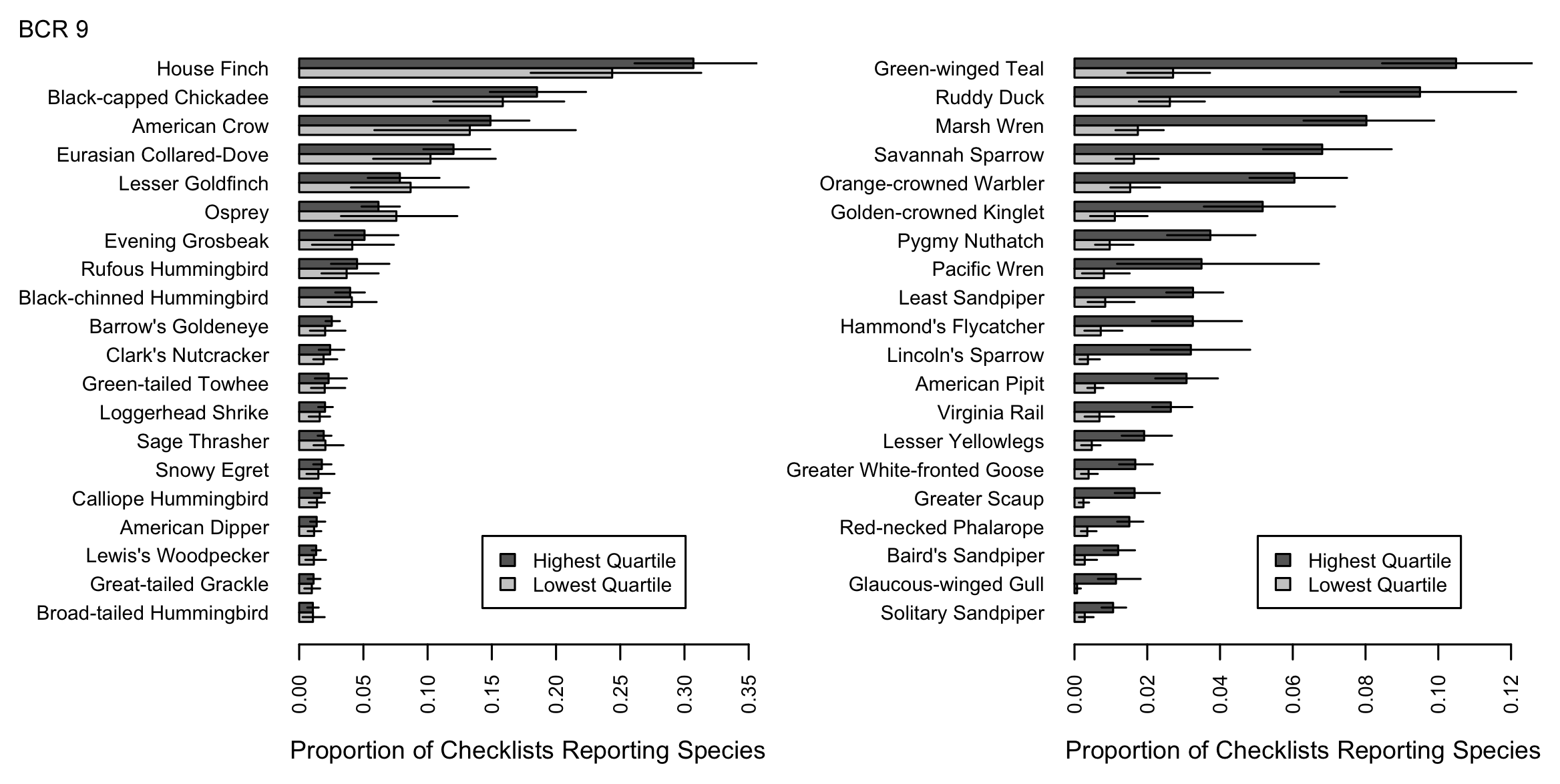

Supplement: S5 Fig — Barplots of the 20 species for which detection rates are proportionally most similar (left) and the 20 species for which detection rates are proportionally most different (right). Detection rate is the proportion of checklists that record a given species and error bars represent 95% bootstrap confidence intervals. The 20 species for which the two groups have proportionally most similar detection rates are generally species that are fairly easy to identify by sight. The 20 species that the two groups have proportionally most different detection rates are generally species that are difficult to identify, easier to identify by sound, or often be seen as a high-flying silhouette without many distinguishing features. (TIF) [file pone.0139600.s005.tif]

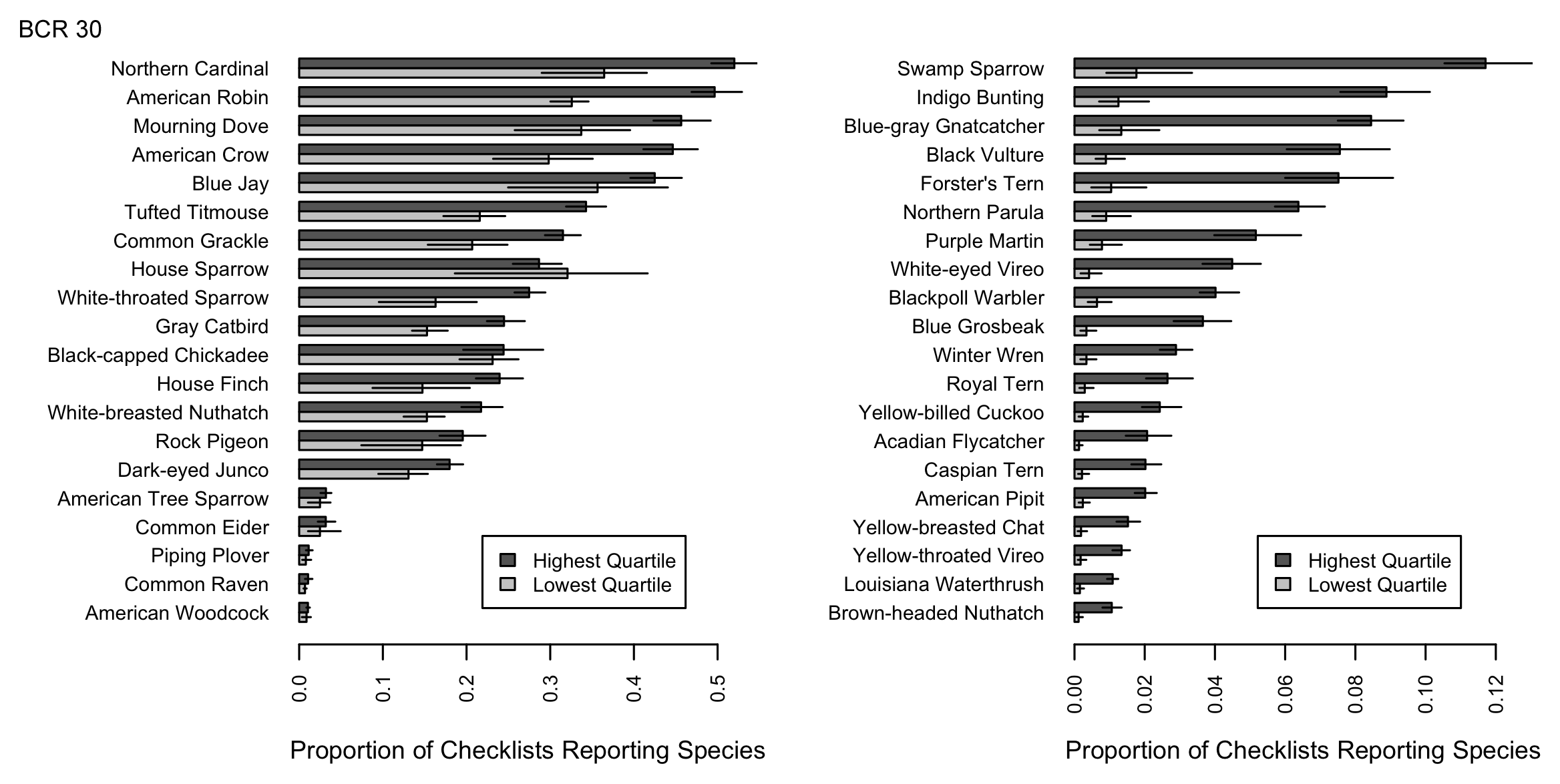

Supplement: S6 Fig — Barplots of the 20 species for which detection rates are proportionally most similar (left) and the 20 species for which detection rates are proportionally most different (right). Detection rate is the proportion of checklists that record a given species and error bars represent 95% bootstrap confidence intervals. The 20 species for which the two groups have proportionally most similar detection rates are generally species that are fairly easy to identify by sight. The 20 species that the two groups have proportionally most different detection rates are generally species that are difficult to identify, easier to identify by sound, or often be seen as a high-flying silhouette without many distinguishing features. (TIF) [file pone.0139600.s006.tif]

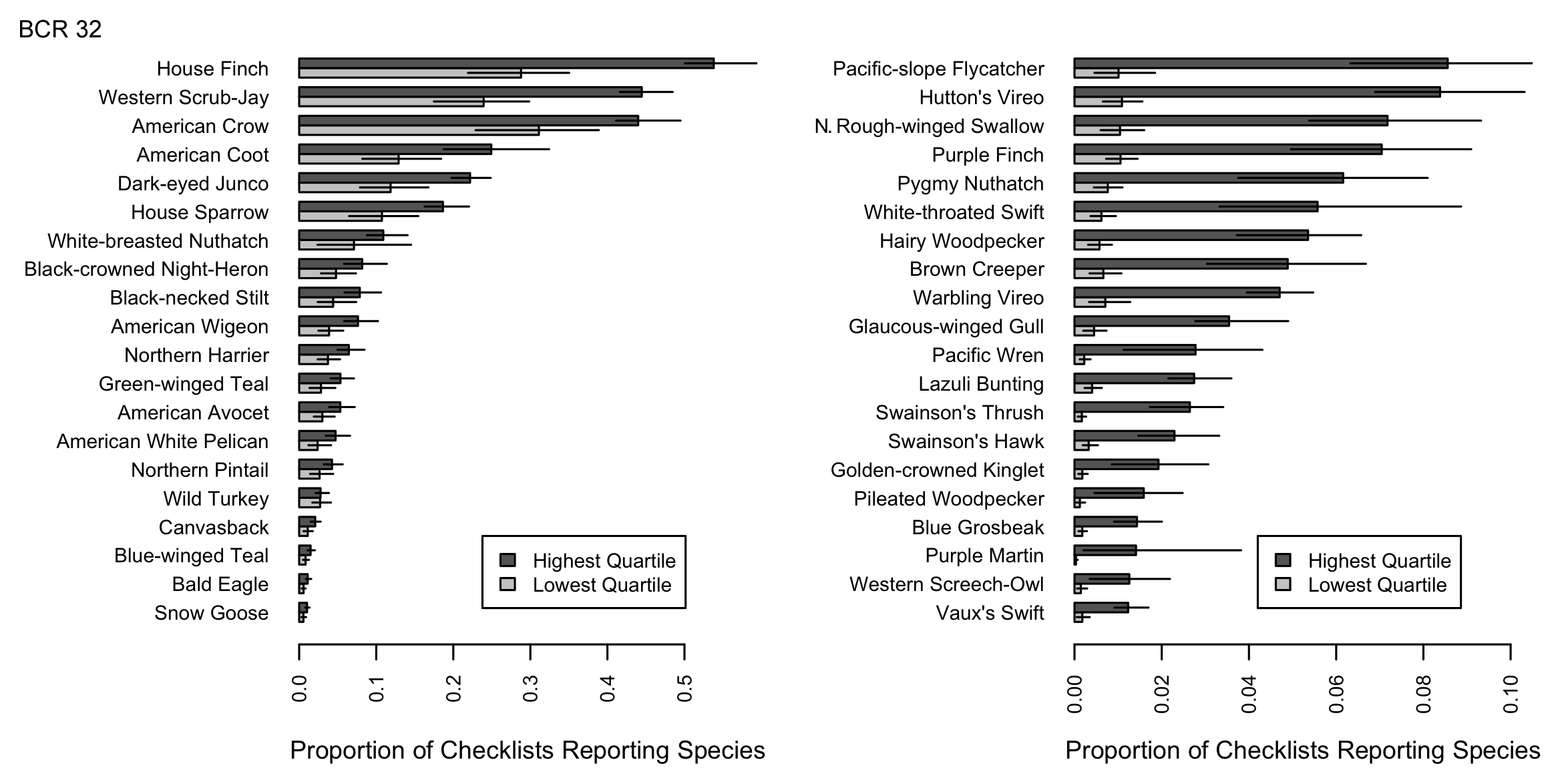

Supplement: S7 Fig — Barplots of the 20 species for which detection rates are proportionally most similar (left) and the 20 species for which detection rates are proportionally most different (right). Detection rate is the proportion of checklists that record a given species and error bars represent 95% bootstrap confidence intervals. The 20 species for which the two groups have proportionally most similar detection rates are generally species that are fairly easy to identify by sight. The 20 species that the two groups have proportionally most different detection rates are generally species that are difficult to identify, easier to identify by sound, or often be seen as a high-flying silhouette without many distinguishing features. (TIF) [file pone.0139600.s007.tif]

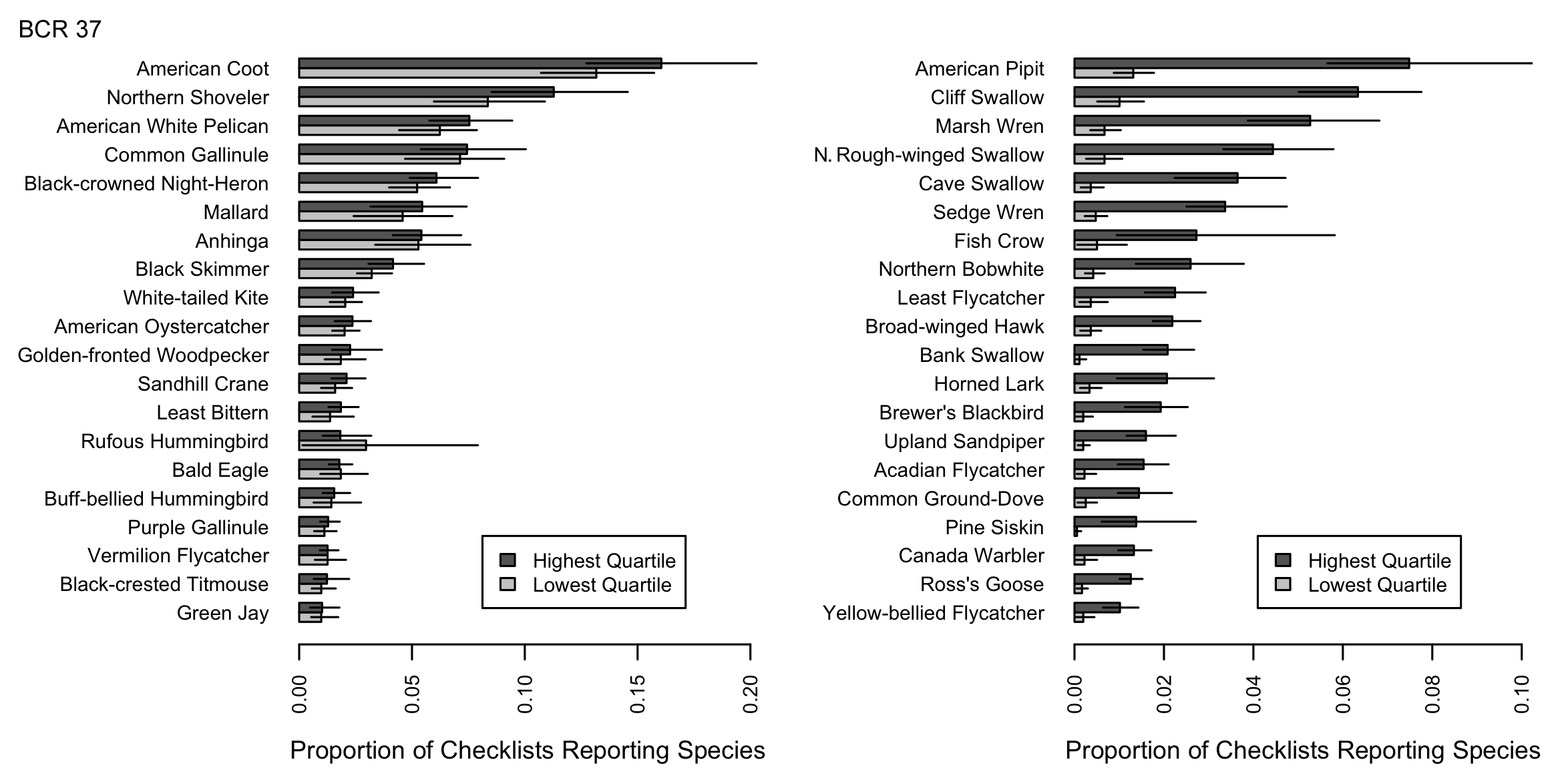

Supplement: S8 Fig — Barplots of the 20 species for which detection rates are proportionally most similar (left) and the 20 species for which detection rates are proportionally most different (right). Detection rate is the proportion of checklists that record a given species and error bars represent 95% bootstrap confidence intervals. The 20 species for which the two groups have proportionally most similar detection rates are generally species that are fairly easy to identify by sight. The 20 species that the two groups have proportionally most different detection rates are generally species that are difficult to identify, easier to identify by sound, or often be seen as a high-flying silhouette without many distinguishing features. (TIF) [file pone.0139600.s008.tif]

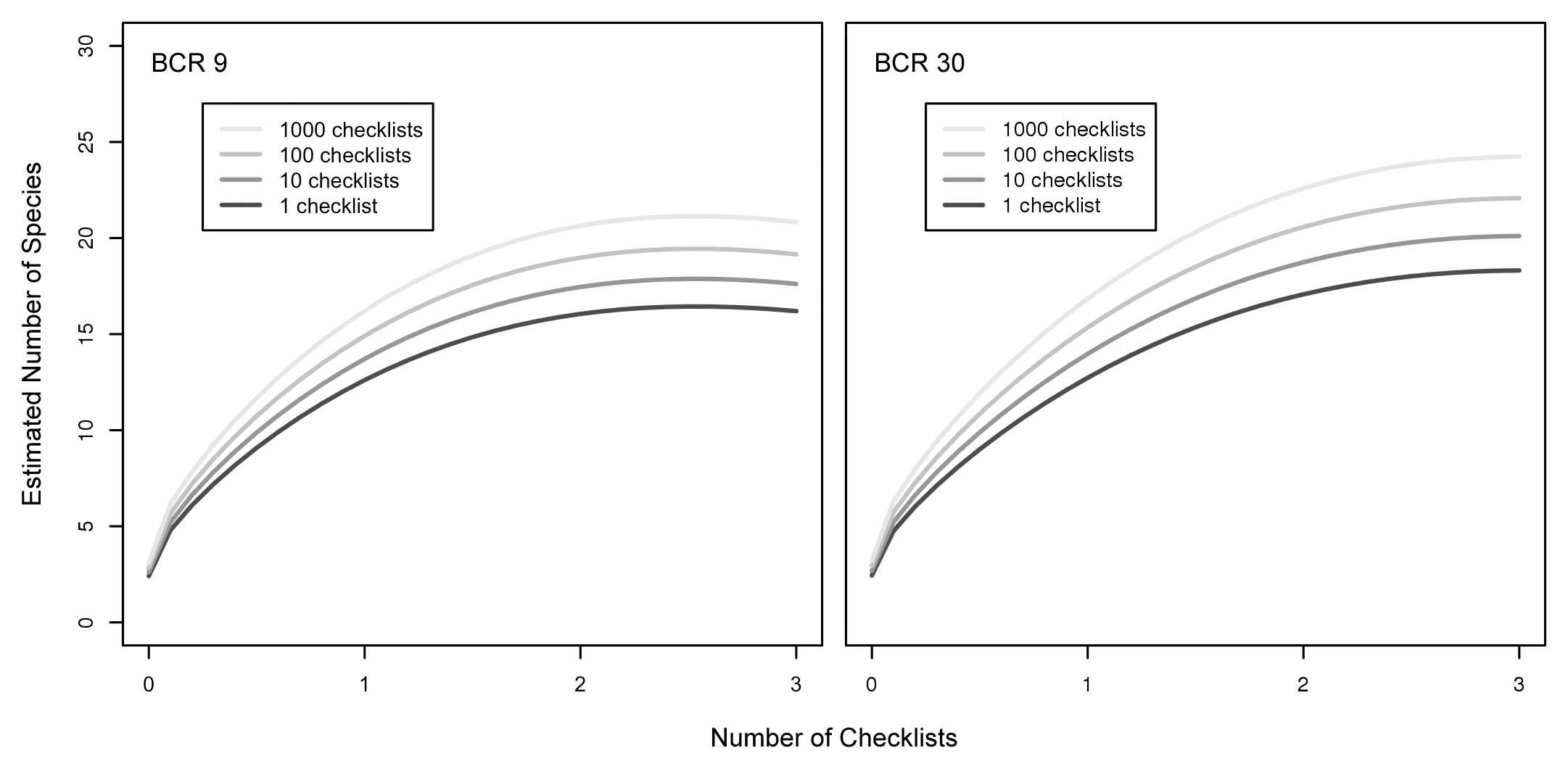

Supplement: S9 Fig — We estimated changes in the number of species observed with increasing number of checklists submitted to eBird, to test whether observers report more species after they have submitted more eBird checklists. To do this we included a covariate of the log of checklist number, which increased sequentially within each observer. Note that while increased participation leads to a higher rate of accumulation of species, this effect is highest for beginning participants and slows with increased participation. (TIF) [file pone.0139600.s009.tif]

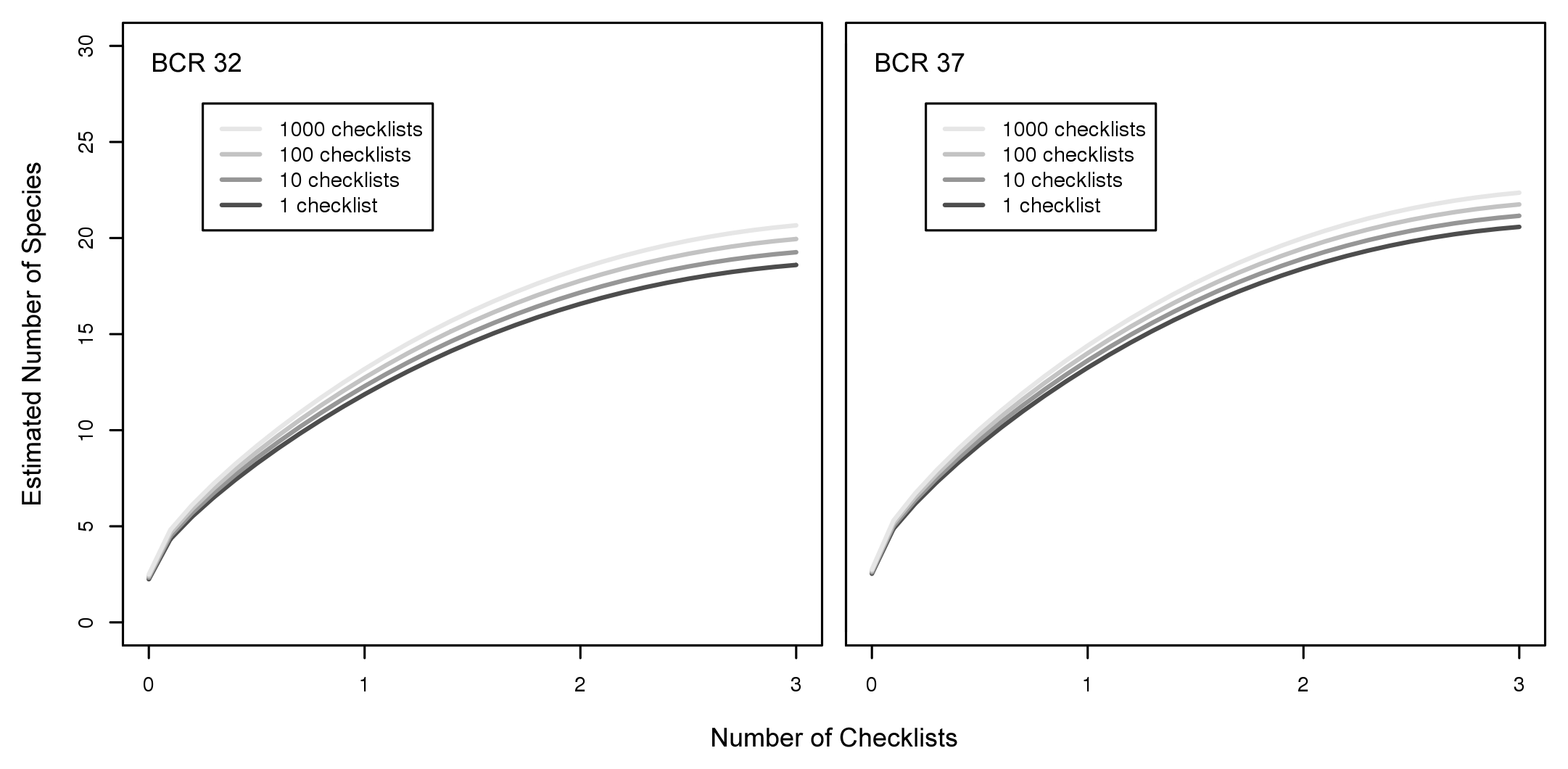

Supplement: S10 Fig — We estimated changes in the number of species observed with increasing number of checklists submitted to eBird, to test whether observers report more species after they have submitted more eBird checklists. To do this we included a covariate of the log of checklist number, which increased sequentially within each observer. Note that while increased participation leads to a higher rate of accumulation of species, this effect is highest for beginning participants and slows with increased participation. (TIF) [file pone.0139600.s010.tif]
